# Captive Common Marmosets (Callithrix jacchus) Are Colonized throughout Their Lives by a Community of *Bifidobacterium* Species with Species-Specific Genomic Content That Can Support Adaptation to Distinct Metabolic Niches

**DOI:** 10.1128/mBio.01153-21

**Published:** 2021-08-03

**Authors:** Lifeng Zhu, Qinnan Yang, Mallory J. Suhr Van Haute, Car Reen Kok, Joao Carlos Gomes-Neto, Natasha Pavlovikj, Resmi Pillai, Rohita Sinha, Haley Hassenstab, Aaryn Mustoe, Etsuko N. Moriyama, Robert Hutkins, Jeffrey French, Andrew K. Benson

**Affiliations:** a Department of Biology, University of Nebraska—Omaha, Omaha, Nebraska, USA; b Nebraska Food for Health Center, University of Nebraska—Lincoln, Lincoln, Nebraska, USA; c Department of Food Science and Technology, University of Nebraska—Lincoln, Lincoln, Nebraska, USA; d School of Biological Sciences, University of Nebraska—Lincoln, Lincoln, Nebraska, USA; e Center for Plant Science Innovation, University of Nebraska—Lincoln, Lincoln, Nebraska, USA; f Department of Computer Science and Engineering, University of Nebraska—Lincoln, Lincoln, Nebraska, USA; Rutgers, The State University of New Jersey

**Keywords:** common marmoset, gum-feeding specialists, *Bifidobacterium*, diet-driven gut microbiomes, gum arabic

## Abstract

The common marmoset (Callithrix jacchus) is an omnivorous New World primate whose diet in the wild includes large amounts of fruit, seeds, flowers, and a variety of lizards and invertebrates. Marmosets also feed heavily on tree gums and exudates, and they have evolved unique morphological and anatomical characteristics to facilitate gum feeding (gummivory). In this study, we characterized the fecal microbiomes of adult and infant animals from a captive population of common marmosets at the Callitrichid Research Center at the University of Nebraska at Omaha under their normal dietary and environmental conditions. The microbiomes of adult animals were dominated by species of *Bifidobacterium*, *Bacteroides*, *Prevotella*, *Phascolarctobacterium*, *Megamonas*, and *Megasphaera*. Culturing and genomic analysis of the *Bifidobacterium* populations from adult animals identified four known marmoset-associated species (*B. reuteri*, *B. aesculapii*, *B. myosotis*, and *B. hapali*) and three unclassified taxa of *Bifidobacterium* that are phylogenetically distinct. Species-specific quantitative PCR (qPCR) confirmed that these same species of *Bifidobacterium* are abundant members of the microbiome throughout the lives of the animals. Genomic loci in each *Bifidobacterium* species encode enzymes to support growth and major marmoset milk oligosaccharides during breastfeeding; however, metabolic islands that can support growth on complex polysaccharide substrates in the diets of captive adults (pectin, xyloglucan, and xylan), including loci in *B. aesculapii* that can support its unique ability to grow on arabinogalactan-rich tree gums, were species-specific.

## INTRODUCTION

Mammalian gut microbiomes play essential roles in host nutrition, immunity, development, and health (1–4). The taxonomic configuration of the gut microbiome is influenced by a number of factors and is highly individualized at the taxonomic ranks of family, genus, and species (5, 6). Complex interactions of host, dietary, and ecological factors influence individuality (7–9), but functional redundancies in metabolic capacities shared across different species of the microbiome may also explain why the functional content of the microbiome is more conserved between individuals than the taxonomic configuration (10).

To facilitate the study of complex interactions that drive the assembly and function of the human microbiome, there is growing interest in New World primates as model systems that are evolutionarily related to humans. Members of the Callitrichidae, such as the common marmoset (Callithrix jacchus), pygmy marmoset (Cebuella pygmaea), tamarins (*Saguinus* spp.), and Goeldi’s marmoset (Callimico goeldii), are of particular interest due to their small size (weight range, 350 to 450 g), short life span (10 to 12 years), stable social family structure, and the absence of human pathogens (11–13).

Callitrichids are omnivores that preferentially eat large amounts of fruits along with the flowering parts of plants in combination with insects, eggs, and small vertebrates (14). They are also gumnivores, feeding heavily on tree gums, and may spend up to 70% of their time harvesting gums when other food sources are low (15–18). Tree gums and exudates are rich in soluble fibers containing high-molecular-weight galactan or mannan backbones ornamented with arabinans and individual residues of arabinose, xylose, rhamnose, and glucuronic acid (19–21). The gum fibers are largely indigestible in the upper gastrointestinal tract and are believed to be digested and fermented by the colonic microbiota. Many marmoset species have evolved anatomical features (dentition and nail structure) for gouging and scraping trees to facilitate gum exudate production along with evolutionary adaptations of the gastrointestinal tract (elongated colons, enlarged ceca, low-passage-number rates) that presumably facilitate fermentation of the tree gum fibers by the colonic microbiome (22–24). Thus, the colonic microbiome of marmosets may reflect unique metabolic capacities for growth on abundant dietary fibers from fruits and tree gums.

Previous studies of the microbiome from captive populations of infant or adult marmosets have demonstrated that species of *Bifidobacterium* are dominant members of the microbiome and that species isolated from marmosets are phylogenetically distinct from human-associated *Bifidobacterium* spp. (25–33). Several of these marmoset *Bifidobacterium* species (B. aesculapii, B. callitrichos, B. reuteri, B. myosotis, B. tissieri) have been isolated from infant or adult marmosets in multiple facilities (31), suggesting that they may play important roles in the life histories of the animals in captivity as well as in the wild. In humans, different sets of *Bifidobacterium* species appear to play distinct roles at different life stages. During infancy, a small number of *Bifidobacterium* species (B. longum subsp. *infantis*, B. bifidum, B. breve) typically dominate the microbiomes of breast-fed infants due to their unique capacity to degrade human milk oligosaccharides (HMO), and these organisms are believed to play important roles in microbiota assembly and function early in life (34–37). During weaning and transition to a complex diet, the human *Bifidobacterium* population declines to 1 to 5% of the total microbiome but also diversifies to include species such as B. adolescentis, B. longum subsp. *longum*, B. catenulatum, and B. pseudocatenulatum (35), which appear to colonize and displace the infant-associated species (38, 39). In the current study, we show that *Bifidobacterium* species which dominate the microbiomes of infant marmosets are resilient and remain dominant members of the microbiota throughout the life history of the host. Genomic analysis suggests that species-specific patterns of metabolic adaptations in these organisms may facilitate growth on major substrates in the captive marmoset host.

## RESULTS

### Features of the fecal microbiome of the captive marmoset population at the Callitrichid Research Center (CRC) at the University of Nebraska at Omaha (UNO).

**FIG 1:**
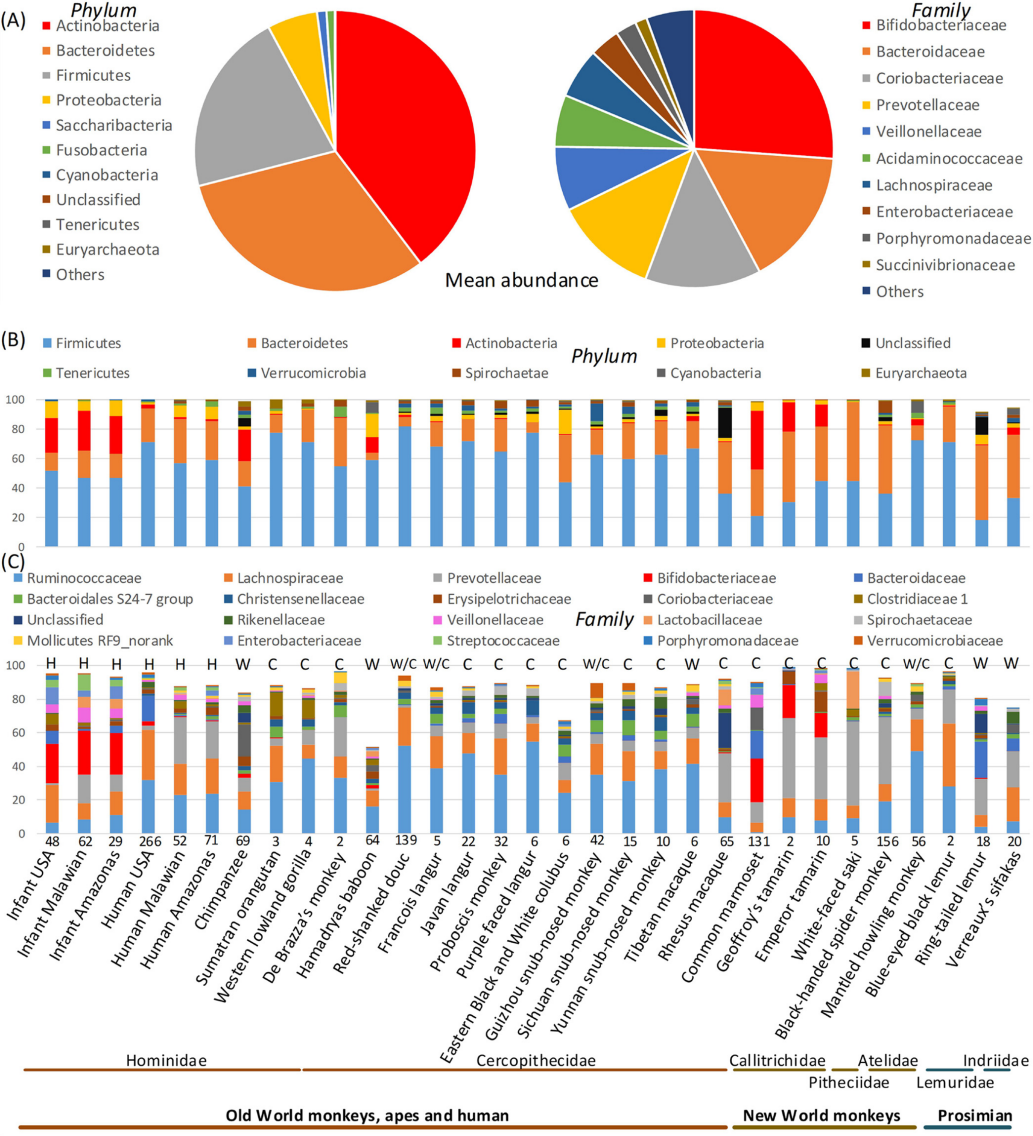
High relative abundances of *Bifidobacteriaceae* and *Bacteroidaceae* in adult common marmoset gut microbiomes. (A) Mean relative abundances of common marmoset gut microbiomes at the phylum level (left) and family level (right). Mean relative abundances of dominant species shared across gut microbiomes of 26 primate species at the phylum (B) and family (C) levels. The sample origins are indicated at the bottom of panel C, with numbers of samples indicated immediately above the name and the sources of the samples indicated above each bar for samples from humans, captive primates, wild primates, and a combination of captive and wild primates (H, C, W, and C/W, respectively). Only dominant taxa shared across most samples are indicated, and nonshared or nondominant taxa account for the remaining relative abundances that total 100%.

The baseline compositions of the gut microbiomes of adult animals under their standard feeding conditions were determined from cross-sectional sampling of 24 adult animals over a 12-week period (131 samples total) (see Table S1A in the supplemental material). Collectively, the microbiotas of animals from this facility were dominated by *Actinobacteria* (40% ± 11.7%), *Bacteroidetes* (31% ± 11.1%), *Firmicutes* (21% ± 7.4%), and *Proteobacteria* (5% ± 2%) (Fig. 1A). Within these phyla, nearly 75% of the collective reads belonged to genera from the families *Bifidobacteriaceae* (26%), *Bacteroidaceae* (16%), *Coriobacteriaceae* (13%), *Prevotellaceae* (12%), and *Veillonellaceae* (7%) (Fig. 1A). Dominant genera included *Bifidobacterium*, *Bacteroides*, *Collinsella*, *Prevotella*, *Alloprevotella*, *Parabacteroides*, *Phascolarctobacterium*, *Megamonas*, *Megasphaera*, and Escherichia (Table S1B). In the majority of the animals, *Bifidobacterium* was the most abundant genus (range, 6 to 64%; median, 26%), and this characteristic has been reported in several other captive marmoset populations (31, 40–43).

10.1128/mBio.01153-21.6 TABLE S1 Sample information in this study. (A) Detailed information of the 131 common marmoset fecal samples collected from 24 common marmosets. (B) Dominant genera in the gut microbiomes of 131 common marmoset fecal samples in this study. (C) Meta-analysis of microbiomes of marmosets and other primates; information is from the 1,418 fecal samples analyzed from 26 primate species. Download Table S1, DOCX file, 0.05 MB. Copyright © 2021 Zhu et al. 2021 Zhu et al. https://creativecommons.org/licenses/by/4.0/ This content is distributed under the terms of the Creative Commons Attribution 4.0 International license.

Meta-analysis of the taxonomic configuration of the microbiomes from CRC marmosets was conducted with 1,287 additional samples from 25 other primate species (Table S1C). With all samples normalized to 13,000 reads per sample, we observed that microbiomes dominated by *Bifidobacterium* were a distinct feature shared by common marmosets, other callitrichids, (Geoffroy’s tamarin and Emperor tamarin), and human infants (Fig. 1 and Fig. S1A). Hierarchical clustering based on Bray-Curtis distances showed that the microbiomes of the adult common marmosets from the CRC and tamarins are more closely related to those of human infants than to those of other primates (Fig. S1B and S1C), and partitioning around medoids (PAM) analysis placed the marmoset and human infant microbiomes in the same medoid (cluster), driven largely by shared high-level abundances of species of *Bifidobacterium*.

10.1128/mBio.01153-21.1 FIG S1 Gut microbiome analysis in the fecal samples of marmosets and other primates. (A) Gut microbiome composition in the fecal samples of marmosets and other primates. Dominant taxa present in the gut microbiome of 26 primate species are indicated with their mean relative abundances (bars) and standard deviation (whiskers). The top panel shows taxon mean abundances in captive marmosets (C), wild marmosets (W), and humans (H). In middle panels and bottom panel, mean abundances of *Bifidobacterium* (B) and *Bacteroides* (C) spp. are shown for the same samples in the top panel. Numbers on the *x* axis indicate the number of samples in the data set. (B) Hierarchical clustering of genus abundances, representing 1,418 fecal microbiome samples from 26 primate species. Clustering was based on the Bray-Curtis distance (using genus relative abundances per sample) among 1,418 fecal microbiomes. Amz, Amazonas; Mal, Malawian. (C) Partitioning around medoids (PAM) clustering algorithm of genus abundances from 1,418 fecal samples from 26 primate species. (D) Dominant *Bifidobacterium* OTUs in adult common marmosets and human infant gut microbiomes. Representative reads of V4 16S rDNA amplicons from OTUs assigned to the genus *Bifidobacterium* by QIIME 2 were used in BLAST searches to identify potential species. The top BLAST hits for species/subspecies of *Bifidobacterium* are indicated at the left for each OTU. No species are listed for OTUs lacking significant hit patterns to known *Bifidobacterium* species. The OTUs were subjected to heatmapping for their relative abundances in humans or the 131 adult marmoset samples from the CRC corresponding to the legend at the bottom of the figure. Download FIG S1, PDF file, 2.3 MB. Copyright © 2021 Zhu et al. 2021 Zhu et al. https://creativecommons.org/licenses/by/4.0/ This content is distributed under the terms of the Creative Commons Attribution 4.0 International license.

### Abundant species of *Bifidobacterium* in the microbiomes of CRC common marmosets.

An initial comparison of the predominant *Bifidobacterium* operational taxonomic units (OTUs) from the adult common marmosets and human infants (Fig. S1D) showed that the OTUs group by host origin, consistent with recent studies describing several new species of *Bifidobacterium* isolated from infant marmosets (25, 27–29, 44). Culture-based enumeration of *Bifidobacterium* cells (serial dilution and plating onto a *Bifidobacterium*-selective medium) from feces of seven different adult animals at three different time points confirmed stable abundances (Fig. S2). Isolates (116 total) from plates of the highest dilutions from seven different animals were chosen and subjected to Sanger-based DNA sequencing of full-length PCR products of the 16S rRNA genes amplified with the primer pair 27F-1392R (45). Among the 72 isolates with >90% identity to known *Bifidobacterium* species (Table S2A), most were highly related (98 to 99% identity) to *B. reuteri*, *B. aesculapii*, *B. callitrichos*, *B. myosotis*, *B. pseudocatenulatum*, *B. hapali*, or B. longum. Eight additional isolates had the best hits (99%) to 16S rRNA gene sequences from one of three distinct *Bifidobacterium* strains (MRM 6.22, MRM 8.12, and MRM 9.26) previously isolated from infant marmosets from a captive population in Italy but which are yet unclassified (Table S2A) (27).

10.1128/mBio.01153-21.2 FIG S2 Plate count enumeration of total *Bifidobacterium* spp. from seven adult marmosets at three time points over a 13-day window. Fecal samples were diluted and plated in triplicate onto *Bifidobacterium* selective medium for each time point, and organisms were enumerated. Averages of the triplicate counts from each time point are plotted on log_10_ scale (*y* axis), with the time points (dates) corresponding to the three different colors. Download FIG S2, DOCX file, 0.02 MB. Copyright © 2021 Zhu et al. 2021 Zhu et al. https://creativecommons.org/licenses/by/4.0/ This content is distributed under the terms of the Creative Commons Attribution 4.0 International license.

10.1128/mBio.01153-21.7 TABLE S2 *Bifidobacterium* analysis in this study. (A) 16S rRNA NCBI BLAST results from 116 isolates obtained from common marmoset fecal samples. (B) Whole-genome sequencing information for 18 *Bifidobacterium* strains isolated from 7 adult common marmosets. (C) The assembly and annotation information of 18 marmoset adult-origin *Bifidobacterium* strains. (D) qPCR primers for marmoset *Bifidobacterium* species. (E) Published *Bifidobacterium* genomes used in this study. Download Table S2, DOCX file, 0.1 MB. Copyright © 2021 Zhu et al. 2021 Zhu et al. https://creativecommons.org/licenses/by/4.0/ This content is distributed under the terms of the Creative Commons Attribution 4.0 International license.

Species identification of *Bifidobacterium* isolates based on 16S rRNA sequences alone is challenging due to the range of variation known to exist within many species (46). Consequently, we used whole-genome sequencing (WGS) to gain further insight into the taxonomic and functional characteristics of the CRC marmoset-derived *Bifidobacterium* isolates. Eighteen isolates were selected for sequencing based on representation of genetic diversity (estimated from full-length 16S rRNA sequences) and diversity in the genders and origins of the animals (Table S2B and S2C).

**FIG 2:**
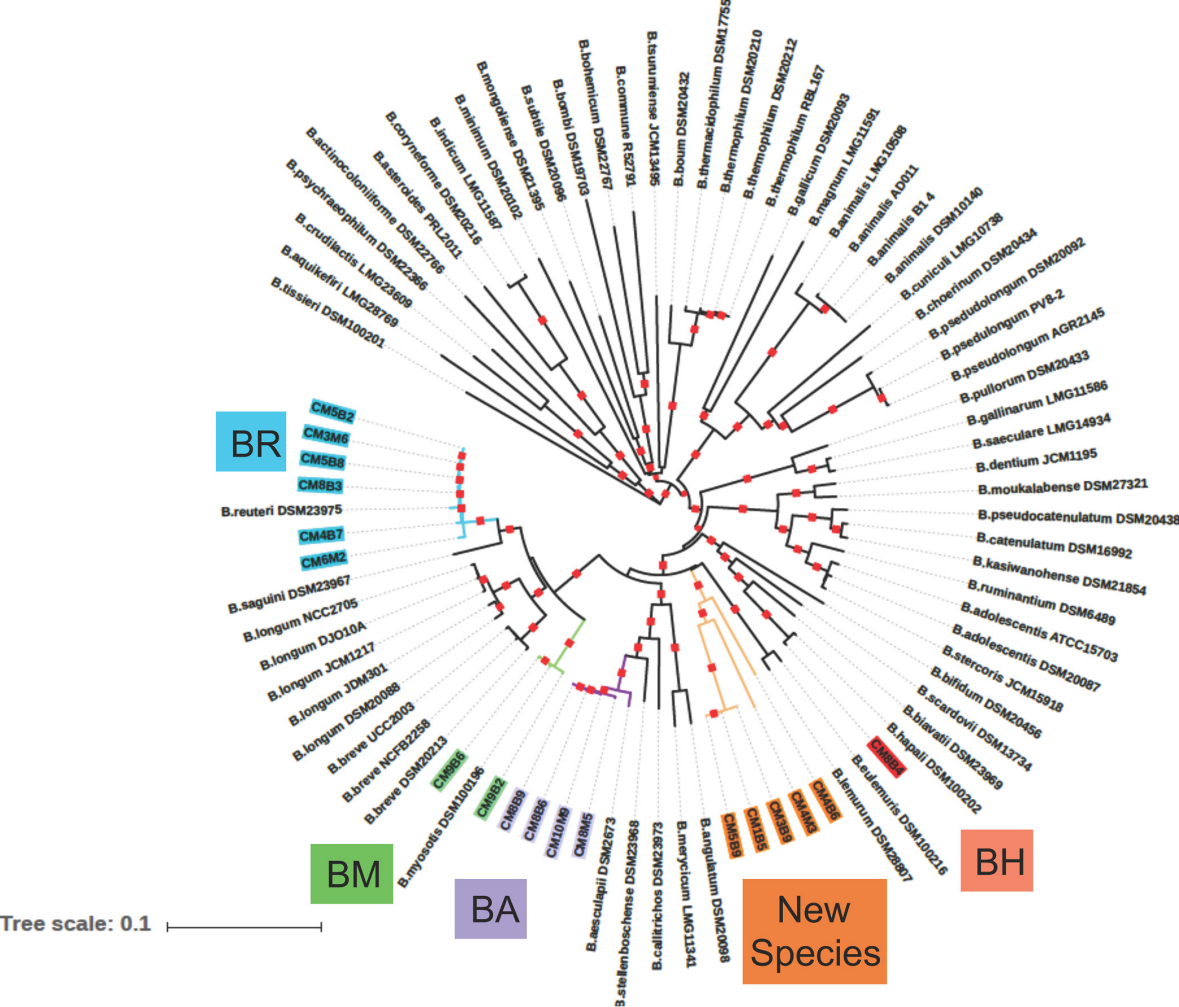
Circular phylogram of the cgMLST-based neighbor-joining phylogenies of common marmoset *Bifidobacterium* isolates. Whole-genome sequencing data were used to define a set of 318 core genes shared by 18 different isolates of *Bifidobacterium* from the common marmosets, along with genomes from 64 other strains representing 48 different species of *Bifidobacterium* from humans, primates, and other host species. The core shared genome from this data set was developed from alignments with MUSCLE (120) (bootstrap, 500), and cgMLST analysis was performed on the concatenated core gene sets by neighbor-joining analysis using FastTree (115). The phylogeny was visualized in iTree of Life; red squares on branches indicate >90% bootstrap values. Isolates from the common marmosets that were assigned to known taxa are color-coded in blue (BR, *B. reuteri*), green (BM, *B. myosotis*), purple (BA, *B. aesculapii*), and red (BH, *B. hapali*), and those assigned as putative new species are in orange (new species MARM_A1, MARM_A2, and MARM_A3).

For taxonomic assignment, genetic distances were computed from a concatenated core genome of 318 genes shared by our 18 isolates and 64 reference strains representing 48 known species of *Bifidobacterium* (Table S2E). A neighbor-joining phylogeny based on pairwise distances is shown in Fig. 2. Of our 18 marmoset isolates, 13 were in clusters supported by significant bootstrap values (<90%) with known species. Based on these metrics, isolates CM5B2, CM3M6, CM5B8, CM8B3, CM4B7, and CM6M2 were assigned as *B. reuteri*, isolates CM9B6 and CM9B2 were assigned as *B. myosotis*, isolates CM8B9, CM8B6, CM10M9, and CM8M5 were assigned as *B. aesculapii*, and isolate CM8B4 was assigned as *B. hapali*.

Core genomes from the remaining 5 of our 18 marmoset-derived isolates (CM5B9, CM1B5, CM3B9, CM4M3, and CM4B6) formed a bootstrap-significant cluster of their own (shown in orange in Fig. 2) that did not include a known species. Three of these isolates (CM5B9, CM1B5, and CM3B9) are highly related to one another and are referred to herein as a singular taxon (MARM_A1), while each of the other two genomes were sufficiently divergent to warrant designation as individual taxa. They are referred to herein as MARM_A2 (isolate CM4M3) and MARM_A3 (isolate CM4B6). Based on genetic distances, it appears that the MARM_A1, MARM_A2, and MARM_A3 taxa represent three distinct species of *Bifidobacterium*.

### *Bifidobacterium* species are a dominant feature of the common marmoset microbiome throughout their lives.

**FIG 3:**
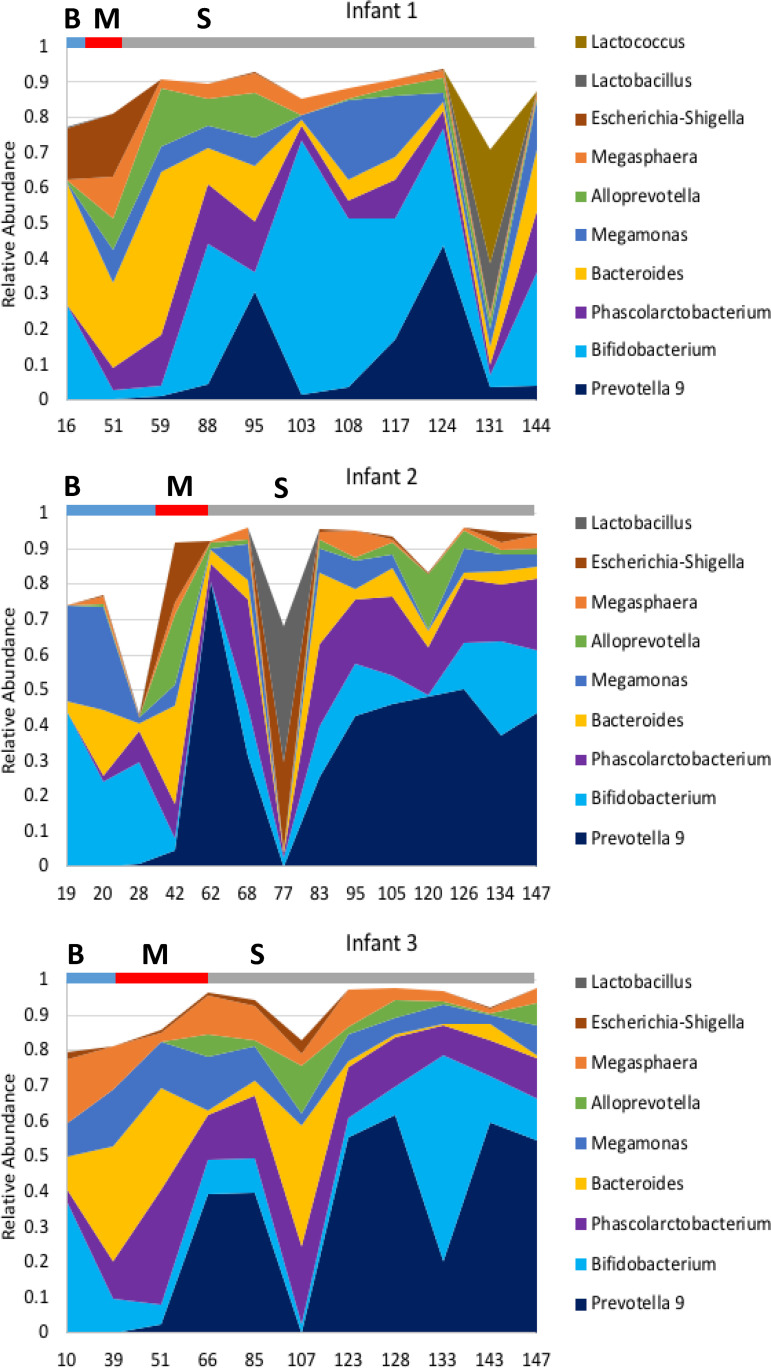
Relative abundances of the dominant genera from 16S rRNA sequencing of the fecal microbiotas of maturing infant marmosets. Area plots are shown for three infants originating from three independent families of common marmosets from the CRC. Sampling time points are indicated in days on the *x* axis, and the relative times that each animal spent exclusively on breastmilk (B, blue bar), mixed breastmilk plus solid food (M, red bar), and solid food (S, gray bar) are indicated by the corresponding lengths of the bars.

Many of the *Bifidobacterium* species isolated from adult animals at the UNO CRC have also been isolated from infant marmosets at other facilities (26–32, 47); however, the *Bifidobacterium* community and its individual species have not been studied systematically and quantitatively throughout the life histories of animals at any facility. We therefore examined the *Bifidobacterium* community and individual species throughout the life histories of the animals using a combination of 16S rRNA amplicon sequencing and species/phylotype-specific quantitative PCR (qPCR) assays. In a pilot longitudinal study with a cohort of three infant animals (each from a different family), microbiome analyses were performed on fecal samples from each infant collected during breastfeeding at day 10, 16, or 19 after birth (blue bars labeled “B” in Fig. 3), during the transition period in which infants are weaning and their diets are mixed at days 43 to 60 after birth (red bars labeled “M” in Fig. 3), and at >60 days after birth, when the animals are fully weaned and exclusively consuming the complex solid diet (gray bars labeled “S” in Fig. 3). During the weaning (M) period, the infants are still breastfeeding, but a complex solid diet is being introduced to the infants by the parents (approximately 15 g of ZuPreem, 5 g of egg, 1.5 g of melon, 1 g of apple, 1 g of meal worms, 1 g of gum arabic, and 8 g of applesauce), whereas fully weaned animals (S) exclusively eat the complex solid diet.

As illustrated in Fig. 3, *Bifidobacterium* spp. were abundant during the B, M, and S phases despite temporal fluxes in the relative abundances. Three additional taxa (*Prevotella*, *Alloprevotella*, and *Phascolarctobacterium*) had notable temporal changes, being undetectable or in very low abundance in samples from the breastfeeding phase but becoming prominent in the microbiotas of all three infants after weaning (Fig. 3). Such patterns suggest that invasions and/or successions in the microbiota may be associated with maturation and/or dietary transition. Despite these putative invasions/successions, the *Bifidobacterium* populations remained abundant members of the microbiomes of all three infants, ranging from 11 to 32% of the microbiota on day 144 for infant 1 and on day 147 for infants 2 and 3.

A more detailed longitudinal study was conducted with a second cohort of infants comprising seven newborn infants: three pairs of twins originating from different dams and a seventh newborn animal from a fourth dam. Fecal samples were collected sequentially from each animal over a 100-day period beginning at approximately 5 to 14 days after birth. Taxon-specific qPCR assays (genus, species, and phylotype levels [see Materials and Methods and Table S2D]) were used to quantify the total *Bifidobacterium* population, the populations of the individual species (*B. aesculapii*, *B. reuteri*, *B. myosotis*, *B. hapali*, and MARM_A1, MARM_A2, and MARM_A3), and populations of two distinct subtypes of *B. reuteri* (*B. reuteri* phylotype 1 and *B. reuteri* phylotype 2).

**FIG 4:**
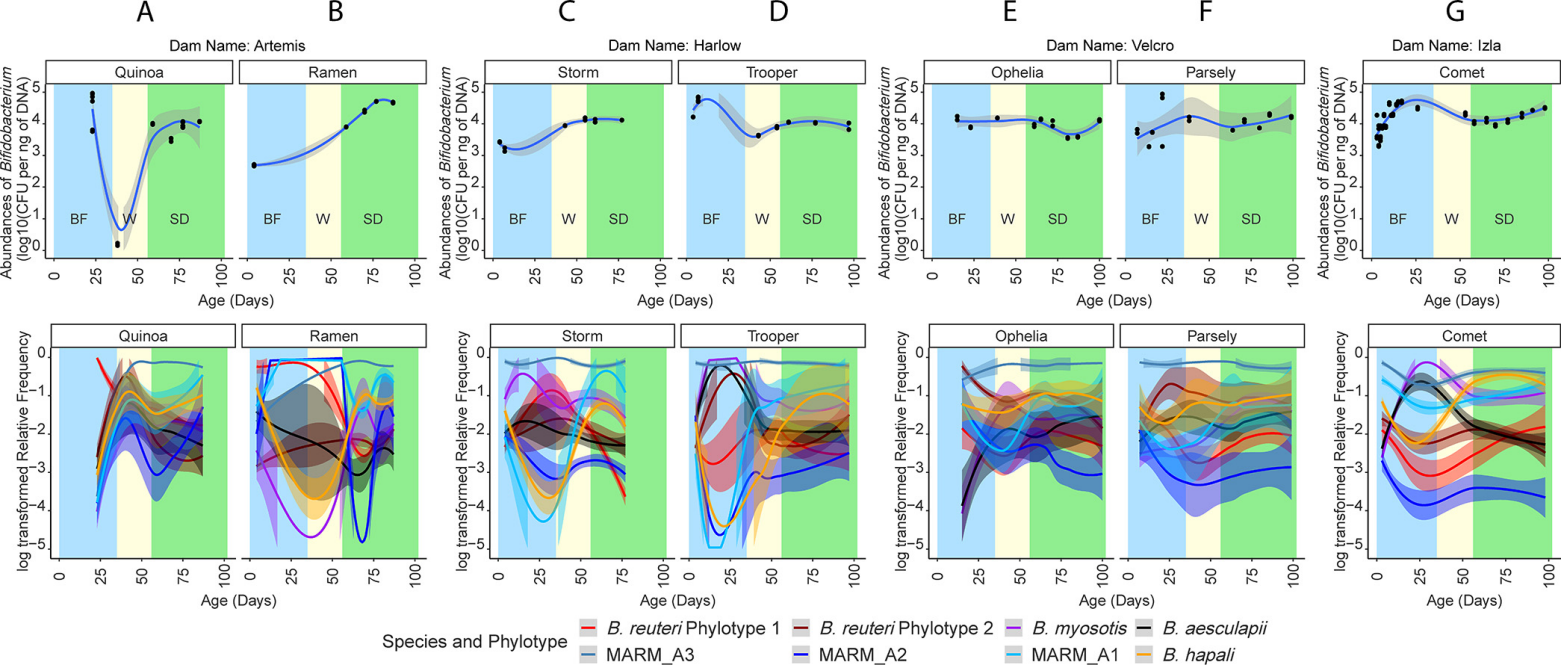
qPCR analysis of *Bifidobacterium* species and subtypes during maturation of a cohort of infant marmosets. A cohort including seven newborn infants was developed from three pairs of twins originating from different dams and from a seventh newborn animal from a fourth dam. (A to G) Data from individual animals. In each panel, the top graph depicts quantification of the total *Bifidobacterium* population, and the lower panel shows quantifications of the individual *Bifidobacterium* species/phylotypes. In each panel, shading is used to show the developmental phase of the individual samples (light blue, the breastfeeding phase; light yellow, weaning; light green, solid diet). Numbers of days after birth are indicated on the *x* axis. Animals from the same dams are shown in panels A and B (dam = Artemis), panels C and D (dam = Harlow), and panels E and F (dam = Velcro), and an individual animal is shown in panel G from dam Izla. The qPCR assays were performed with genus-specific primers for total *Bifidobacterium* spp. and with primers specific for individual species/phylotypes (*B. reuteri* phylotype 1, *B. reuteri* phylotype 2, *B. myosotis*, *B. aesculapii*, *B. hapali*, MARM_A1, MARM_A2, and MARM_A3). Trend lines for data from each species and subtype are color-coded according to the key. The values for total *Bifidobacterium* spp. are reported as log_10_ numbers of CFU per nanogram of total DNA, while the log-transformed relative frequencies are reported for each species and phylotype by dividing the log_10_ number of CFU per nanogram of DNA for each species/phylotype by the sum of log_10_ number of CFU per nanogram of total DNA from all detectible species/phylotypes from the same sample. Trendlines of the relative frequencies were generated by LOWESS, with variance depicted by shading of the trendlines.

The qPCR assays for the total *Bifidobacterium* population were normalized to the total DNA content of the samples to account for different volumes/densities of fecal matter collected from the infants versus from older animals. The qPCR data (Fig. 4, top panels) showed some differences in total *Bifidobacterium* levels between the B and S phases, but after weaning, the population was present at relatively consistent levels across all animals. To quantify the individual species and phylotypes, plots of the species- and phylotype-specific qPCR data (Fig. 4, bottom panels) were developed by normalizing the log_10_ number of CFU per nanogram of DNA for each species/phylotype by the sum of log_10_ numbers of CFU per nanogram of DNA of all species/phylotypes at each time point. Despite temporal variation in the proportions of the different species/phylotypes, the most important new finding was that each of the species and phylotypes was detected at all time points in all animals. Thus, these *Bifidobacterium* species/phylotypes colonize the animals early in life and persist throughout the life histories of the animals.

**FIG 5:**
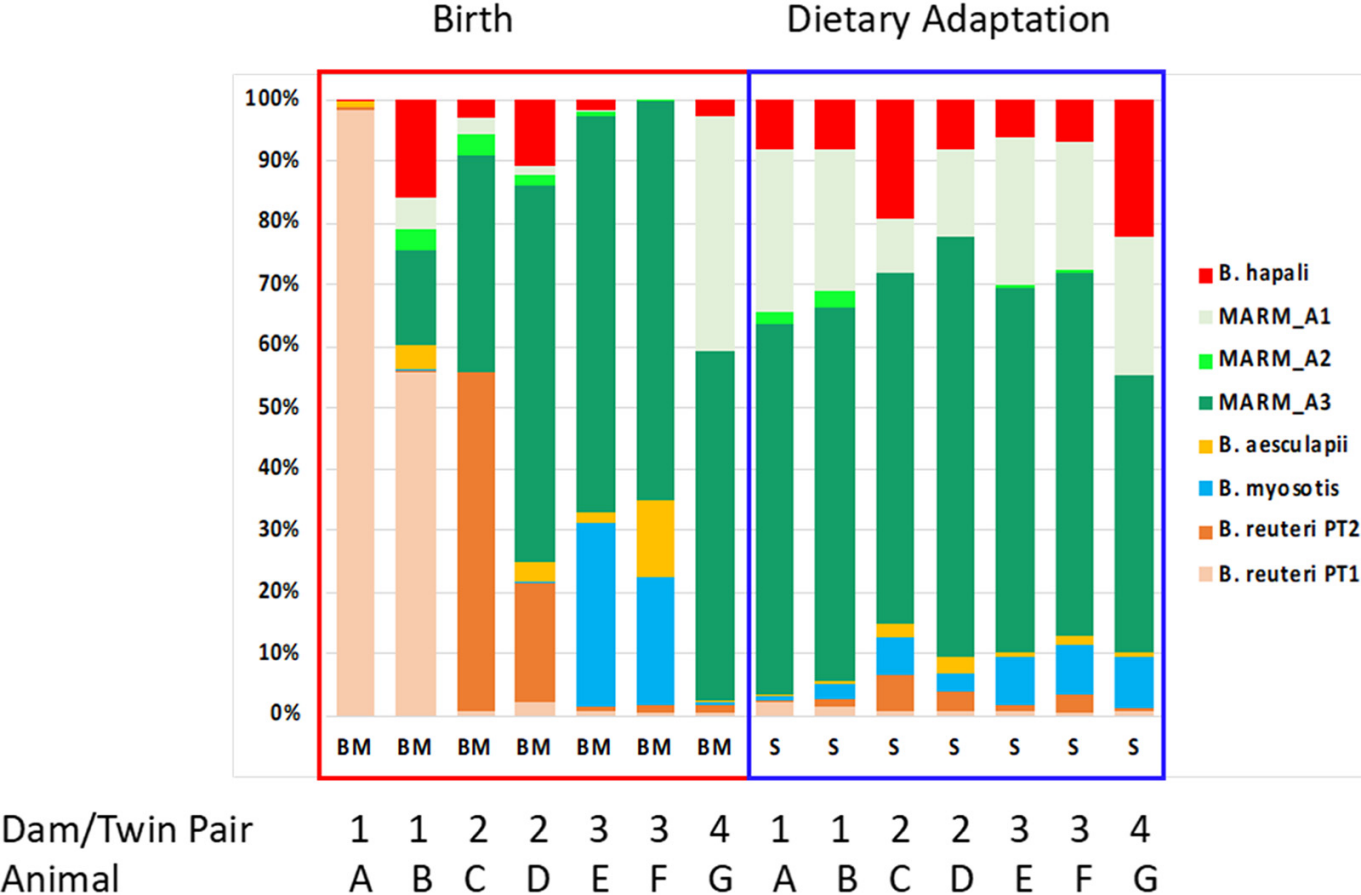
qPCR-based relative proportions of *Bifidobacterium* species and phylotypes from twin pairs converge after adaptation to a complex diet. Bar charts show the percent abundance of each *Bifidobacterium* species and phylotype in twin pairs from the earliest time point after birth (left panel, outlined in red) and the latest time points after dietary adaptation (right panel, outlined in blue). The percent abundances were calculated from the qPCR-based relative frequencies shown in Fig. 4 and standardized to a total combined abundance of 100% for all eight taxa (*B. reuteri* phylotype 1 [PT1], *B. reuteri* phylotype 2 [PT2], *B. myosotis*, *B. aesculapii*, *B. hapali*, MARM_A1, MARM_A2, and MARM_A3). Twin pairs from the same dam are indicated by numbers on the *x* axis, while the alphabetic letters identify individual animals on the birth and dietary adaptation panels.

Temporal variation in the relative proportions of *Bifidobacterium* species was greatest during the breastfeeding and weaning stages, and animal-animal variation also appeared to be greater during the breastfeeding/weaning stages than during the latest time points after dietary adaptation (Fig. 4 and 5). Statistical analysis of the animal-animal variation by one-way analysis of variance (ANOVA) of Bray-Curtiss distances of the *Bifidobacterium* species abundances at the earliest time point in breastfeeding (BM) and terminal time point after adaptation to the complex diet (S) confirmed that indeed much greater variation was detected during the breastfeeding stage (*P* < 0.001). Thus, there is individuality in start points for relative proportions of *Bifidobacterium* species, but maturation and adaptation to the complex diet appear to drive the convergence of the relative proportions of *Bifidobacterium* spp. to a consistent pattern across animals, where the MARM_A3, MARM_A1, and *B. hapali* species become dominant members of the *Bifidobacterium* population.

### Marmoset-associated *Bifidobacterium* species encode enzymatic potential for growth on the major breast milk oligosaccharides from marmosets.

We next searched the genomes of the marmoset *Bifidobacterium* species for metabolic capacities that would enable growth on dietary substrates at the different stages of life. As in humans, neonatal marmosets obtain most of their nutrition from breastmilk. In human breastfed infants, a small number of *Bifidobacterium* species (B. longum subsp. *infantis*, *B. bifidum*, and *B. breve*) have a competitive advantage due to their unique enzymatic capacities for degrading specific oligosaccharides that are found among an ornate array of human breastmilk oligosaccharides (34–37), and it is likely that similar metabolic capacity is important for neonatal colonization in marmosets. Elegant, comparative studies of the breastmilk glycome have shown that marmoset breastmilk contains two major oligosaccharides, lacto-*N*-tetrose (LNT), which is also abundant in humans, and lacto-*N*-neohexose (LNnH) (48). We therefore searched the genomes of the marmoset *Bifidobacterium* spp. for orthologous genes that comprise key points of the pathways that have been defined in organisms such as B. longum and *B. bifidum* (36, 49–52).

**FIG 6:**
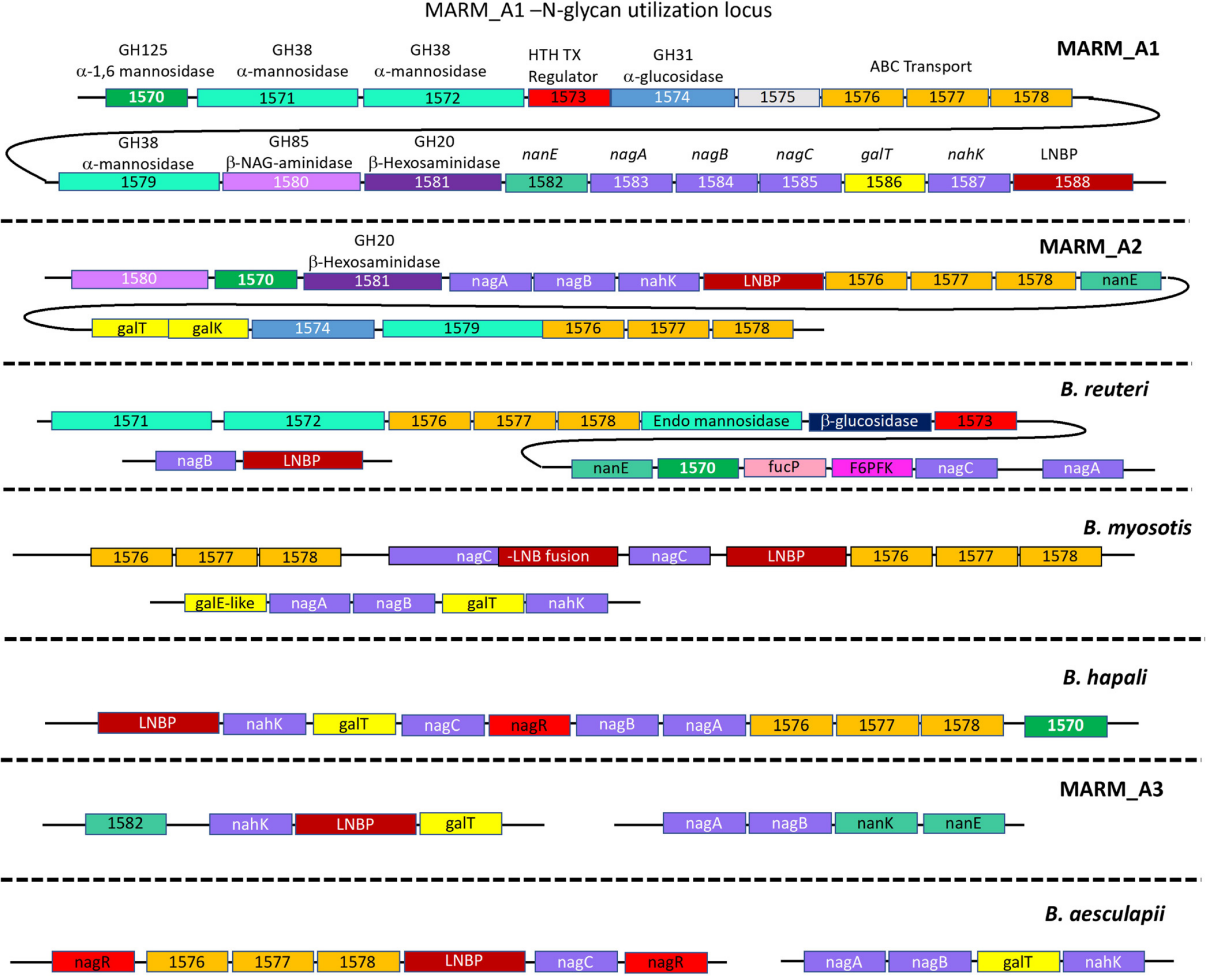
Comparative genomic analysis of candidate genes for degradation of marmoset breastmilk oligosaccharides. Putative orthologues for lacto-*N*-biose phosphorylase (LNBP) and *N*-acetylglucosamine utilization were identified from the genomes of marmoset-derived *Bifidobacterium* species and are depicted in their genomic contexts for each of the species. Curved lines denote linked sets of genes. The relevant gene content for each species is separated by dotted lines. The LNBP gene (maroon) and adjacent genes encoding α-mannosidases (green), α-glucosidases (blue), transporters (orange), *N*-acetyl hexosaminidase (purple), *N*-acetylglucosamine utilization (light purple), α-fucosidases (pink), and galactose utilization (yellow) are shown with gene ID numbers for orthologous genes corresponding to the MARM_A1 annotation. HTH TX, helix-turn-helix transcription factor.

Among the 18 genomes of our marmoset *Bifidobacterium* species/strains, each carried orthologous genes for LNT degradation, including a 1,3-β-galactosyl-*N*-acetylhexosamine phosphorylase (LNB phosphorylase [LNBP]) gene and one or more β-galactosidase genes orthologous to the GH42 type 1 HMO-specific β-galactosidase III gene at the Blon_2106 locus of B. longum (Fig. 6). All strains also carried intact *nagA* and *nagB* genes (Fig. 6), which would enable *N*-acetyl-galactosamine derived from cleavage by type I HMO-specific β-galactosidases to be metabolized through a pathway similar to that of B. longum (deacylation by NagA [E.C. 3.5.1.25; Blon_0882], deamination to fructose 6-phosphate by NagB [E.C. 3.5.99.6; Blon_0881], and subsequent metabolism through the central bifid shunt pathway). Genomic contexts of the LNBP, *nagA*, and *nagB* genes in the CRC marmoset *Bifidobacterium* taxa divides these organisms into two major groups (Fig. 6). The first group (MARM_A1, MARM_A2, and *B. reuteri*) carry the LNBP gene, *nagB*, and/or *nagA*, within putative N-glycan islands. In the MARM_A1 and MARM_A2 genomes, *nagA*, *nagB*, and the LNBP gene are parts of islands that encode one or more α-mannosidases (GH125 family and GH38 family), GH31 α-glucosidases, GH85 β-*N*-acetyl-galactosaminidases, and GH20 β-hexosaminidases. *B. reuteri* carries the *nagA* gene in the context of an N-glycan island (Fig. 6) with a unique combination of GH38 and GH125 α-mannosidases, a GH3 family β-glucosidase, and a *fucP*-like α-fucosidase. The N-glycan island (and genome) of this organism lacks the GH85 β-*N*-acetylglucosamine amindase and the GH20 β-hexoseaminidase genes, and the LNBP and *nagB* genes are found at separate loci. If these organisms can indeed grow on LNT, genomic architecture embedding of LNBP, *nagA*, and/or *nagB* in islands with mannosidases, hexosaminidases, glucosidases, and fucosidases in MARM_A1, MARM_A2, and *B. reuteri* imply that the enzymatic capacity for LNT degradation may have evolved from the genomic capacity for the degradation of N-glycans. The GH20 β-hexoseaminidases in the islands of MARM_A1 and MARM_A2 may also catalyze the cleavage of the β-1,6-*N*-acetylglucosamine-galactose bond in the other major marmoset breastmilk oligosaccharide (LNnH), potentially enabling MARM_A1 and MARM_A2 to grow on both of the dominant marmoset breastmilk oligosaccharides (LNT and LNnH). The presence of the unique α-fucosidase gene *B. reuteri* island may also enable this organism to grow on marmoset milk oligosaccharides with α-fucosyl linkages.

The second group of marmoset *Bifidobacterium* species (*B. myosotis*, *B. hapali*, *B. aesculapii*, and MARM_A3) also carry type I HMO-specific β-galactosidases, LNBP, *nagA*, and *nagB* genes, which may promote growth on LNT (Fig. 6). However, in this group of taxa, N-glycan islands were not detected in their genomes, nor were any genes encoding β-1,6-hexosaminidases detected. Consequently, we hypothesize that the growth of *B. myosotis*, *B. hapali*, *B. aesculapii*, and MARM_A3 on milk oligosaccharides may be restricted to LNT.

### Accessory genomes of marmoset-derived *Bifidobacterium* species encode a species-specific enzymatic and metabolic capacity for adaptation to different components of the captive-marmoset diet.

Accessory genomes of *Bifidobacterium* species (and/or strains) often carry species-specific or subtype-specific combinations of enzymatic capacity that can facilitate growth on plant-derived storage polysaccharides or cell wall glycans (53). Thus, we examined the genomes of the marmoset *Bifidobacterium* species to identify genes that could facilitate growth on complex carbohydrates that are enriched in the captive-marmoset diet. Fruit makes up 30 to 40% of the base captive-marmoset diet, followed by the wheat bran and soy meal components of ZuPreem. Comparative genomic analyses subsequently identified unique metabolic islands in the accessory genomes of these organisms encoding the enzymatic capacity for degrading pectins, xyloglucans, and xylans, which are major components of dietary fruit (pectins and xyloglucans), wheat bran (xylans and arabinoxylans), and soy meal (pectins).

**FIG 7:**
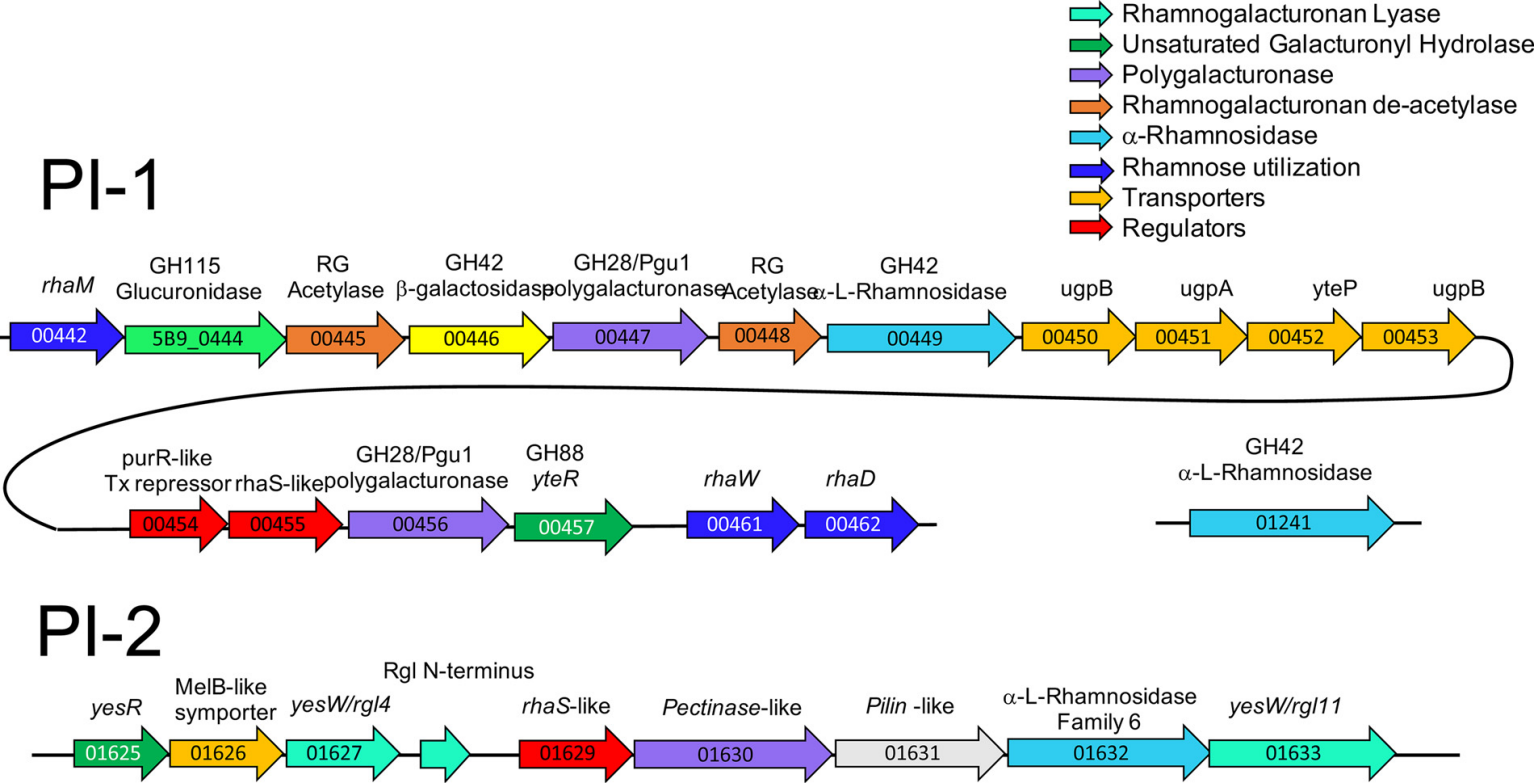
Gene islands for pectin utilization in MARM_A1. The two islands, termed pectin island 1 (PI-1) and pectin island 2 (PI-2) are shown with the relevant functional contents of the genes in each island color-coded according to the key in the upper right corner. The MARM_A1 gene IDs are indicated for each gene, and the curved line in PI-1 denotes contiguous genes. The 01241 α-rhamnosidase gene is positioned at a site distal from PI-1 and PI-2.

The MARM_A1 genome was the most distinctive, with two metabolic islands encoding an enzymatic machinery for nearly complete disassembly of pectins and a third island encoding genes for the degradation of xyloglucans. Predicted enzymes in pectin islands 1 and 2 (PI-1 and PI-2, respectively) (Fig. 7) include pectinases, polygalacturonases, rhamnogalactan lyases, unsaturated galacturonyl hydrolases, rhamnogalacturon deacetylases, and rhamnosidases, which collectively promote complete disassembly of the polygalacturonic acid backbone in homogalacturonans and rhamnogalacturonan II, as well as enzymes for cleaving the alternating α-galacturonic acid–α-rhamnose backbone found in rhamnogalacturonan I. Genes encoding a GH42 family β-galactosidase and a GH115 family β-glucuronidase are also present in PI-1, which enables cleavage of galactose side chains and glucuronic acid decorations. MARM_A1 also encodes two genes for the central metabolism of rhamnose at the PI-1 locus (*rhaW*, encoding l-rhamnoate dehydratase, and *rhaD*, encoding 2-keto-3-deoxy-l-rhamnoate aldolase). Rhamnose utilization (53) and pectin degradation (54, 55) are relatively rare traits among *Bifidobacterium* species, with the notable exception of the pectin-degrading capacity found in B. vespertilionis isolated from fruit bats (56). Although PI-1 and PI-2 were absent in the MARM_A3 genome, *rhaW* and *rhaD* were found adjacent to a 6×H family α-l-rhamnosidase, suggesting that this organism may cross-feed from the cleavage of rhamnose residues from the major pectin backbones by MARM_A1.

Complementing the PI-1 and PI-2 islands for pectin degradation in MARM_A1 is a third island for xyloglucan degradation (XG-1) (Fig. S3). Xyloglucans (XGs) are also major components of hemicelluloses in fruit that cross-link cellulose from cell walls of adjacent cells, and fruit-encoded enzymes play important roles in fruit ripening by catalyzing partial degradation/disassembly of XGs (57). The MARM_A1 XG-1 island (Fig. S3) includes genes encoding β-glucosidases, α-xylosidases, and α-fucosidases, which may promote complete disassembly of the β-1,4-linked glucosyl backbone and side chains with α-1,6-linked xylose, β-1,2-galactose, and α-1,2-fucose XGs. XG-1 islands were also present in the genomes of *B. myosotis* and *B. hapali* (Fig. S3) and, along with the α-l-rhamnosidase/rhamnose utilization system found in MARM_A3, may enable MARM_A3, *B. myosotis*, and *B. hapali* to work in concert with MARM_A1 to attack the major pectin fibers and cell-cell cross-linking xyloglucans in dietary fruit.

10.1128/mBio.01153-21.3 FIG S3 Xyloglucan islands present in the genomes of MARM_A1, *B. myosotis*, and *B. hapali*. Genomic islands that encode putative enzymes for the degradation of xyloglucans are aligned from the genomes of MARM_A1, *B. myosotis*, and *B. hapali*. Genes encoding a putative ABC transport system for galacturonooligosaccharides are shaded in orange, including the *yteP*-like gene at the 5′ end. Putative esterase genes are colored light blue, the *afuC*-like α-fucosidases are colored red, GH3 *bglX*-like β-glucosidases are dark blue, GH5 *bglC*-like β-endoglucanases are blue, GH31 α-xylosidase is light green, GH43 β-xylosidase is dark green, and GH42 β-galactanase is yellow. Download FIG S3, DOCX file, 0.1 MB. Copyright © 2021 Zhu et al. 2021 Zhu et al. https://creativecommons.org/licenses/by/4.0/ This content is distributed under the terms of the Creative Commons Attribution 4.0 International license.

A novel metabolic feature of the phylotype 1 subtype of *B. reuteri* is predicted by a large xylan metabolic island (XI-1) with enzymatic content to support the degradation of xylans and arabinoxylans from wheat bran present in the ZuPreem captive-marmoset diet (Fig. S4). XI-1 is distinct from the plant oligosaccharide degradation locus found in B. longum (58), and it likely facilitates the extracellular degradation of the xylan backbone to β-xylooligosaccharides (β-XOS) through a putative Sec1-secreted GH10 XynA-like endo-β-xylanase (5B2_00301) that includes a C-terminal carbohydrate binding domain (CBM9_1) common to endo-hydrolyzing β-xylanases (59). Four different genes encoding intracellular β-xylosidases are also present in XI-1, three of which are members of the well-characterized class of GH43 family β-xylosidases/α-l-arabinases (>70% identity); the fourth encodes a GH8 family β-exo-oligoxylanase with 54% identity to a characterized enzyme from Bacillus halodurans (60).

10.1128/mBio.01153-21.4 FIG S4 XL-1 xylan island unique to the genome of *B. reuteri* phylotype 1 strains. The XL-1 xylan island from *B. reuteri* phylotype 1 strains is shown at the top. The island is inserted adjacent to the xylose isomerase gene, and the corresponding regions adjacent to the xylose isomerase gene are indicated below from the genomes of *B. myosotis*, *B. reuteri* phylotype 2, *B. hapali*, *B. aesculapii*, MARM_A2, MARM_A1, and MARM_A3. Genes in the *B. reuteri* phylotype 1 XL-1 island include *ugpB*, *ugpA*, and *ugpE*-like genes, encoding components of a putative ABC transport system (orange), genes with GH domains characteristic of β-xylanases, β-xylosidases, and α-arabinases (shades of green), and the GH2-containing xylan esterase like gene (gray). Xylan-associated genes at this same position in the *B. myosotis* genome encode two GH43-domain XynB-like xylosidases/arabinases (shades of green), an AES-type esterase (light blue), and a putative acetyltransferase (blue). Download FIG S4, DOCX file, 0.1 MB. Copyright © 2021 Zhu et al. 2021 Zhu et al. https://creativecommons.org/licenses/by/4.0/ This content is distributed under the terms of the Creative Commons Attribution 4.0 International license.

### Daily consumption of gum arabic in captive animals may be important for preserving gum-degrading species in the microbiome.

At the CRC, animals are given tree gum (gum arabic) on a daily basis at 0.5 g per day per cage. While this daily dose is relatively low in proportion to other complex dietary carbohydrate substrates, daily exposure to the gum, which shares structural features of the galactan backbones (β-1,3-linked galactose chains with β-1,6-linked galactose side chains) with other galactan-rich tree gums (61, 62), may be extremely important for preserving gum-degrading microbes that may play important roles in the microbiomes of wild animals.

**FIG 8:**
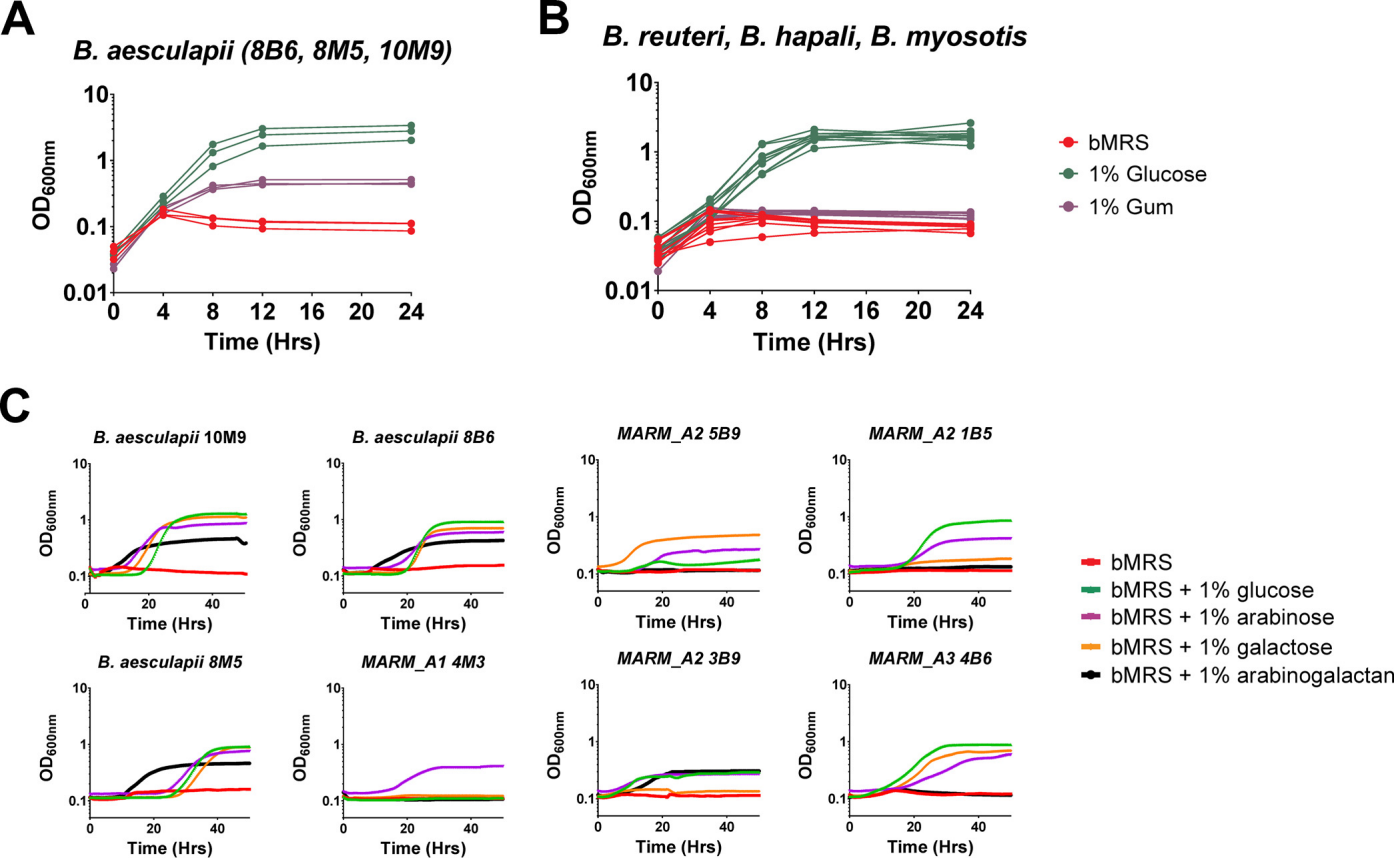
Growth of *Bifidobacterium* isolates on gum arabic and arabinogalactan. (A and B) Liquid cultures of the isolates were grown on 1% glucose, 1% gum arabic, or bMRS. The graph in panel A is from the three isolates of *B. aesculapii* (8B6, 8M5, 10M9), while panel B shows data from isolates of *B. reuteri*, *B. myosotis*, *B. hapali*, MARM_A1, MARM_A2, and MARM_A3. OD_600_ readings were taken at 0, 4, 8, 12, and 24 h of anaerobic culture at 37°C. (C) The *B. aesculapii* isolates were grown in liquid cultures of bMRS containing 1% glucose, 1% arabinose, 1% galactose, 1% larch wood arabinogalactan, or bMRS.

Growth on arabinogalactans (AGs) is relatively rare among *Bifidobacterium* species (63–65), prompting us to compare the abilities of marmoset *Bifidobacterium* species to grow in monocultures on gum arabic as a primary carbon source. Overnight cultures of the strains were grown anaerobically in De Man, Rogosa, and Sharpe (MRS) medium plus 1% glucose and then inoculated (1:100 dilution) into fresh basal MRS medium (bMRS) and bMRS supplemented with either 1% gum arabic or 1% glucose. The cultures were grown anaerobically at 37°C, with samples removed periodically to measure optical density (OD) (Fig. 8A and B). The *Bifidobacterium* strains grew well on glucose under these conditions, but only the three isolates of *B. aesculapii* (8B6, 8M5, and 10M9) showed measurable growth on gum arabic (Fig. 8A). Within the 24-h growth period, the *B. aesculapii* isolates grown on gum arabic achieved about half the density of growth on glucose, but their apparent growth rates on gum arabic or glucose during logarithmic growth phase were quite similar.

Subsequent testing of the marmoset *Bifidobacterium* species for growth on Larchwood arabinogalactan was done using overnight anaerobic cultures (bMRS with 1% glucose) diluted into fresh bMRS alone or bMRS containing 1% arabinogalactan, 1% galactose, 1% arabinose, or 1% glucose. As shown in Fig. 8C, the patterns of growth on Larchwood arabinogalactan were essentially identical to growth on gum arabic; only the three strains of *B. aesculapii* (8B6, 10M9, and 8M5) grew appreciably on Larchwood arabinogalactan, their apparent log-phase growth rates on the Larchwood arabinogalactan and glucose were similar, and the cultures reached about half the density of those grown on glucose.

Among tree species that are preferentially targeted for gum harvest by wild populations of Callithrix jacchus (66–68), gums from preferred species, such as Anadenanthera colubrina and Anacardium occidentale, comprise arabinogalactans with β-1,3-linked galactan backbones and β-1,6-galactosyl side chains that are structurally similar to those of gum arabic (19, 69). Thus, the unique ability of *B. aesculapii* to grow efficiently on gum arabic and Larchwood arabinogalactan illustrates the potential for this organism to play critical metabolic and ecological roles in the microbiome and the need to include gum arabic in the diets of captive animals.

### Genomic loci predicted to support the growth of *B. aesculapii* suggest adaptation to unique metabolic niches associated with gummivorous lifestyles.

**FIG 9:**
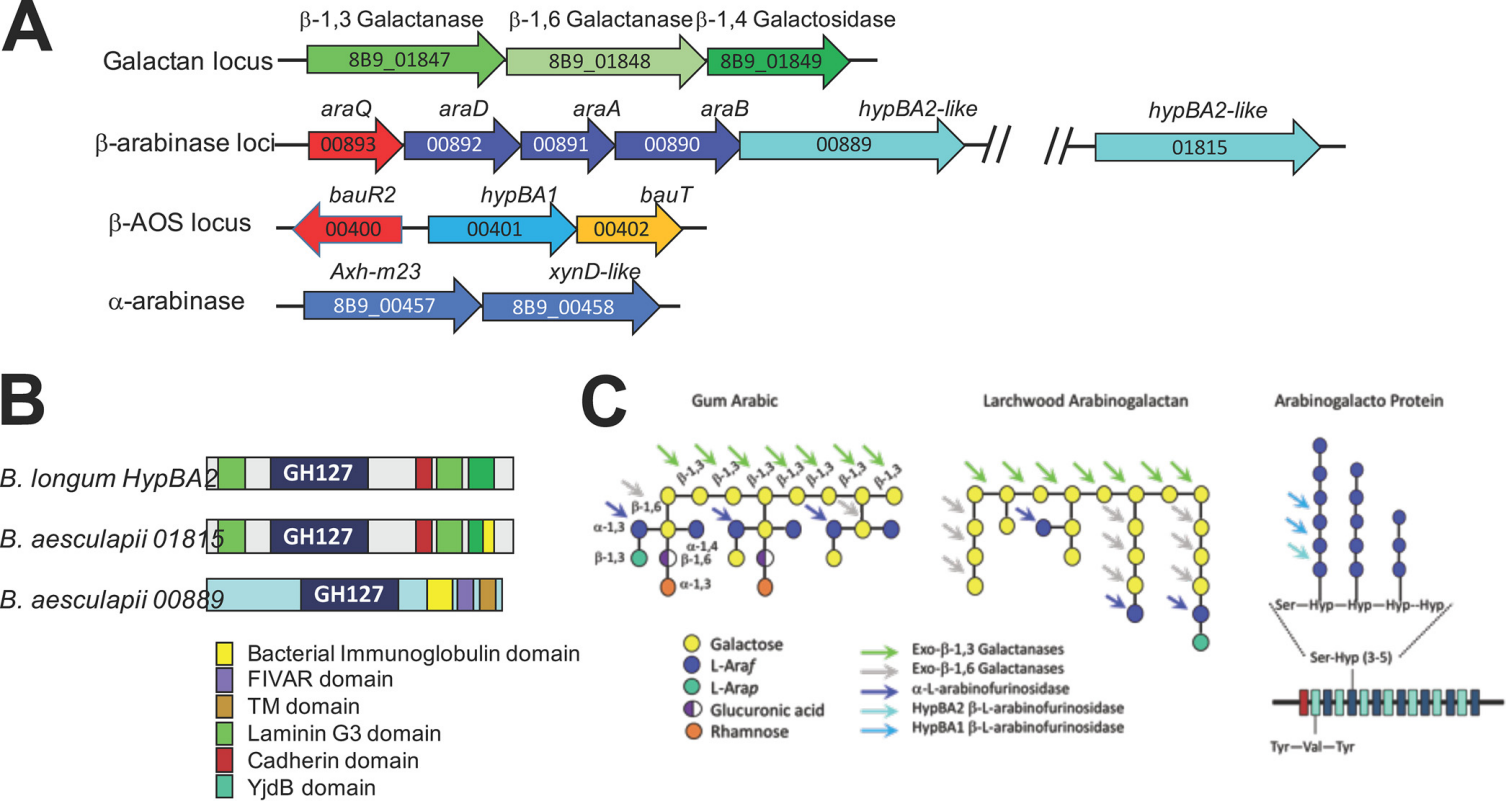
Candidate genomic loci for tree gum degradation in *B. aesculapii.* (A) Sets of genes encoding galactanases and arabinases at four different genomic positions in *B. aesculapii*. Genes are colored according to their predicted activities (green = galactanases, blue = arabinases, red = transcription regulators, yellow = transport). (B) The domain structures of the GH127 HypBA2 β-arabinose from B. longum is shown along with the putative GH127 HypBA2-like proteins from *B. aesculapii*. Domain colors are defined at the bottom of the panel. (C) Backbone structures of gum arabic, larch wood arabinogalactan, and the ArabinoGalacto protein b extensin. Individual carbohydrate monomers are colored according to the key in the lower left of the panel, and sites for cleavage by different galactanases and arabinases are indicated by the colored arrows.

Based on shared structural similarities of the backbone AGs, β-arabinans, and arabinogalactan proteins (AGPs) in tree gums and gum arabic (61, 70), BLAST analysis with B. longum enzymes having known β-1,3 galactanase, β1,6 galactanase, α-furinosidase, or β-arabinase activities was used to identify candidate orthologues (>70% identity over 80% of the protein or domain length) in *B. aesculapii*. Candidate genes were detected in five different loci in the *B. aesculapii* genome (Fig. 9A) but not in other marmoset *Bifidobacterium* species. These loci are referred to as the β-galactan locus, the α-arabinase locus, the β-arabinooligosaccharide (β-AOS) locus, and two different β-arabinase loci.

Within the β-galactan locus of *B. aesculapii* are two adjacent genes encoding cell surface-localized β-galactanases (Fig. 9A and C). These *B. aesculapii* enzymes (8B9_01847 and 8B9_10848) have >72% identity across their full lengths to enzymes encoded by an adjacent gene pair in B. longum JCM1217 (BLLJ_1840 and BLLJ_1841) with β-1,3 and β-1,6 galactanase activities, respectively (64). The B. longum BLLJ_1840 protein has β-1,3 galactanase activity on arabinogalactans (63) and shares a GH43 exo-β-1,3 galactanase domain along with cell surface localization domains at the C terminus (F5 to F8 and bacterial-immunoglobulin-like domains) with the *B. aesculapii* 8B9_01847 protein. The orthologue of the *B. aesculapii* 8B9_01848 gene in B. longum (BLLJ_1841) encodes a β-1,6-galactobiohydrolase enzyme (64), and the enzymes from both species share linear combination of GH30 glycohydrolase domains, two different high-affinity galactose-binding domains (Gal-binding domain [GBD] and a ricin-like domain) and three C-terminal domain proteins for cell surface localization (an F5-F8 domain and two different bacterial-immunoglobulin domains).

The α-arabinase locus in *B. aesculapii* (Fig. 9A and C) encodes adjacent extracellular enzymes that can catalyze the cleavage of individual α-arabinose residues decorating the galactan backbone of gum arabic and Larchwood arabinogalactans (71–73). The two adjacent α-l-arabinofurinosidases (8B9_00457 and 8B9_00458) have >73% identity to a characterized adjacent gene pair found in B. longum (BL1544 and BL1543). The B. longum BL1544 and *B. aesculapii* 8B9_00457 genes encode secreted s-Axh1 α-l-arabinofurinosidases, and the BL1544 enzyme has known activity on α-1,3- and α-1,2-linked terminal arabinose residues from arabinogalactan (74). The BL1543 gene in B. longum and the 8B9_00458 gene in *B. aesculapii* encode GH43-XynD-like β-xylosidases/α-1,3-arabinofurinosidases with N-terminal (CBM4_9) and C-terminal (CBM6) carbohydrate binding domains, and recombinant proteins derived from BL1543 have α-1,3-arabinofurinosidase activity (74).

Genes encoding extracellular β-arabinases are found in two different loci in the *B. aesculapii* genome (Fig. 9A). Both these β-arabinase loci (8B9_00889 and 8B9_01815 genes) encode proteins with GH127 family β-arabinase domains that are related to the HypBA2 β-l-arabinase from B. longum, which is known to cleave short β-arabinooligosaccharides (β-AOS) from AGPs (75). The predicted protein from 8B9_01815 (Fig. 9B) is structurally orthologous to B. longum HypBA2 (72% identity over its full length), sharing the GH127 domain along with C-terminal laminin G3 and the YjhB-like wall-binding domains of HypBA2. In contrast, the GH127 β-arabinase domain of 8B9_0899 (Fig. 9B) is linked to different C-terminal wall-associated domains (bacterial-immunoglobulin 2 family, found in various architectures [FIVAR], and a transmembrane [TM] domain). However, the 8B9_0899 β-arabinase is positioned as a downstream member of the central *araDBA* arabinose utilization operon in *B. aesculapii*, and remarkably, orthologues of the *B. aesculapii* 8B9_0889 gene are found only in *Bifidobacterium* species isolated from callitrichids (B. primatium, B. parmae, and B. catulorum); in each of these species, the 8B9_0889 β-arabinase orthologues are positioned as members immediately downstream of the central *araDBA* operon (Fig. S3).

To support growth on β-AOS produced by the extracellular β-arabinases, *B. aesculapii* strains also carry orthologues (>72% identity across full-length alignment) of a β-AOS symporter (BauT) and intracellular GH127 family β-arabinase (HypBA1) from B. longum (Fig. 9) that is known to hydrolyze β-AOS2 and β-AOS3 into arabinose monomers (76). Together, the *B. aesculapii* cell surface β-arabinases (8B9_0899 and 8B9_01815), the *bauT*-encoded transporter, and the intracellular *hypBA1*-encoded β-arabinase enable this species to cleave short β-AOS from AGPs outside the cell, transport the β-AOS into the cell, and degrade β-AOS intracellularly into arabinose monomers.

## DISCUSSION

The marmoset is an emerging nonhuman primate model with several characteristics that make it a tractable model for studying the microbiome, and marmoset microbiomes are actively being studied in several different marmoset colonies and wild callitrichid populations (40–43, 77). One of the most distinctive features shared by animals across multiple facilities, including the CRC, is the dominance of their microbiomes by species of *Bifidobacterium* (25–27, 29, 31, 44, 47) (see Fig. S1A in the supplemental material).

At the species level, the exact combinations of *Bifidobacterium* species and their relative proportions are facility specific, but a core set of *Bifidobacterium* species (*B. aesculapii*, *B. reuteri*, *B. myosotis*, and *B. hapali*) have consistently been isolated from independent captive-marmoset facilities. Even the unclassified MARM_A1, MARM_A2, and MARM_A3 found in the CRC animals appear to have been identified in other facilities (27). The core set of *Bifidobacterium* species may be particularly adapted to dietary and other factors associated with captivity, and systematic studies to compare microbiotas from wild and captive callitrichids should therefore be prioritized to provide appropriate physiological and ecological contexts for interpreting characteristics of these species in captive animals.

### Enzymatic capacity for marmoset breastmilk oligosaccharides among the marmoset-derived *Bifidobacterium* species may contribute to colonization early in life.

Our qPCR analysis of the individual *Bifidobacterium* species and phylotypes (*B. aesculapii*, *B. reuteri* phylotype 1, *B. reuteri* phylotype 2, *B. myosotis*, *B. hapali*, MARM_A1, MARM_A2, and MARM_A3) originally isolated from adult animals at the CRC facility showed that each taxon is an abundant member of the microbiota throughout the life histories of the animals (Fig. 4). It is likely that breastmilk oligosaccharides (e.g., LNT and LNnH) are primary substrates for these *Bifidobacterium* species during the breastfeeding stage, and each of the marmoset *Bifidobacterium* species from our study encodes the enzymatic capacity to support growth on LNT (Fig. 6), an abundant oligosaccharide in marmoset breastmilk (48). Candidate genes to facilitate the degradation of LNnH, the other primary marmoset milk oligosaccharide (48), were found only in MARM_A1, MARM_A2, and *B. reuteri* (Fig. 6), suggesting that these species may have the capacity for growth on both LNT and LNnH. It will be important to confirm these activities, to determine if there is differential utilization among these substrates, and to determine whether these organisms utilize strategies similar to those of B. longum subsp. *infantis* (transport and intracellular degradation of the oligosaccharides) or the *B. bifidum* strategy (extracellular degradation) (78).

The LNT and LNnH genes in the MARM_A1, MARM_A2, and *B. reuteri* genomes are embedded in genomic islands that encode α-mannosidases, α-glucosidases, and β-hexosaminidases, which are commonly associated with N-glycan degradation (Fig. 6). Thus, MARM_A1, MARM_A2, and *B. reuteri* may have evolved their capacity for milk oligosaccharide degradation through adaptation of the existing genomic capacity for growth on mucins, which has been hypothesized for human *Bifidobacterium* species (79). In contrast, *B. myosotis*, *B. aesculapii*, *B. hapali*, and MARM_A3 lack N-glycan islands but have acquired the LNBP gene and genes for *N*-acetylglucosamine utilization in species-specific genomic contexts (Fig. 6).

### Resilience of the *Bifidobacterium* population during maturation and adaptation to a complex diet.

Although the species of marmoset bifidobacteria in our studies are lifelong members of the microbiome, temporal fluctuations in the total *Bifidobacterium* population and proportions of different *Bifidobacterium* species were observed, particularly during weaning and dietary adaptation (Fig. 3 and 4). Based on 16S rRNA gene amplicon sequencing (Fig. 3), abundances of *Bifidobacterium* and *Bacteroides* spp. declined during dietary transition, and their decline was accompanied by substantial increases in *Prevotella* and *Phascolarctobacterium* organisms. These taxa were undetectable or in very low abundance during breastfeeding, suggesting that niche opportunity associated with the dietary transition may drive the invasion/expansion of *Prevotella* and *Phascolarctobacterium* populations. Nonetheless, populations of both *Bifidobacterium* and *Bacteroides* appear to be resilient, and they return to nearly pretransition abundance levels after dietary adaptation.

As with the proportions of different genera, proportions of the individual *Bifidobacterium* species also varied measurably over time (Fig. 4). In neonatal animals, the proportions of *Bifidobacterium* species were unique to individuals but converged to a uniform pattern across animals after dietary transition (Fig. 5). The convergence was supported statistically and suggests that individual factors, such as maternal environment and breastmilk production, may impact microbiota configuration early in life but that maturation and/or transition to a complex diet drives a more uniform distribution of the species across the animals. Future studies should be developed to define the relative contributions of diet and developmental maturation to the convergence and to determine how convergence relates to changes in the overall microbiome (e.g., successions and invasion by organisms such as *Prevotella* and *Phascolarctobacterium*).

### Genomic architecture of individual marmoset *Bifidobacterium* species implies species-specific patterns of metabolic adaptation to major components of the captive-marmoset diet.

Comparative genomic analyses of marmoset *Bifidobacterium* spp. identified species-specific genetic capacities for the degradation of abundant components in the captive-marmoset diet, including pectins (Fig. 7), xyloglucans (Fig. S3), and xylans (Fig. S4). Very few species of *Bifidobacterium* are known to possess the metabolic capacity for growth on pectins (54, 55). Thus, the unique combination of PI-1 and PI-2 pectin islands and the and XG-1 xyloglucan island in MARM_A1 supports the hypothesis that MARM_A1 is a keystone species, metabolically adapted to niches created by consumption of fruit. Such adaptation, coupled with the abundance of fruit in the captive-marmoset diet (30 to 40% of the total captive diet), likely explains why this organism is consistently found as one of the most abundant *Bifidobacterium* species in adult animals (Fig. 4 and 5). Pectins and xyloglucans play different structural/functional roles in fruits, and there may be hierarchal patterns to the disassembly of these substrates by the different species. Thus, it will be important to understand how the capabilities of pectin and xyloglucan degradation by MARM_A1, the xyloglucan degradation of *B. myosotis* and *B. hapali*, and the rhamnose utilization of MARM_A3 relate to ecological niches of these *Bifidobacterium* species.

In addition to finding fruit-derived fibers, we found a distinctive metabolic capacity for xylan degradation (XI-1 island) in the genome of B. reuteri phylotype 1 strains (Fig. S4). The gene content of the XI-1 island is quite distinct from those of known arabinoxylan islands found in human *Bifidobacterium* species. The captive diet contains xylans from grains (e.g., wheat bran components in ZuPreem), which are not likely encountered in the wild-animal diet. Thus, understanding the substrate specificities and natural substrates for XI-1 in B. reuteri will help explain its potential metabolic role.

### *B. aesculapii* may be a keystone organism that supports gummivory in wild marmosets and may be preserved by feeding gum arabic to captive animals.

*B. aesculapii* was also metabolically distinct, with a unique capacity for growth on gum arabic (Fig. 8), which is structurally similar to tree gums consumed by marmosets in the wild. *B. aesculapii* isolates carry a combination of genes for degrading components of gum arabic (Fig. 9) and was consistently present as a minor member of the *Bifidobacterium* community (Fig. 4 and 5). It is quite possible that daily exposure to gum arabic in captivity creates a unique metabolic niche for this organism and is therefore important for maintaining this organism as part of the microbiome. In the wild, gummivory is a major aspect of the callitrichid lifestyle, exemplified by anatomical and physiological evolutionary adaptations (23, 80), and *B. aesculapii* may therefore provide a gateway for understanding metabolic and ecological roles of gum-degrading gut microbes in wild animals as well as their contributions to the overall physiology and health of captive animals.

Gum arabic is derived from *Acacia senegal* trees, which are not native to South America, but the AG component of this gum shares the same core backbone structure (β-1,3-linked galactose polymers with β-1,6-galactosyl side chains [61]) with gums of native species in South America that are commonly visited by wild populations of marmosets (19, 69). The core AG backbone of gum arabic is part of a complex proteoglycan superstructure where AG polymers and β-arabinans are covalently joined to repetitive amino acid sequence AGPs (71, 73). While it is not known if the proteoglycan superstructures are conserved in gums from these native species, the *B. aesculapii* genome encodes unique enzymatic machinery for extracellular degradation of both AGs and β-arabinans (Fig. 9), suggesting that this organism is metabolically adapted to both AG and β-arabinan substrates.

Although extracellular β-arabinases were present only in the *B. aesculapii* genome, genomes of each CRC marmoset *Bifidobacterium* species encode one or more intracellular HypBA1-like β-arabinases. Thus, *B. aesculapii* may play a keystone role in the extracellular degradation of β-arabinans from tree gums, producing free β-arabinooligosaccharides (β-AOS) that can cross-feed each of the other *Bifidobacterium* species. In some species, the *hypBA1*-like β-arabinase genes have undergone genomic expansion (three copies in *B. hapali*, MARM_A2, and MARM_A3 and five copies in MARM_A1), suggesting that these organisms may be particularly adapted to cross-feeding on β-AOS produced by *B. aesculapii*.

Further insight into the importance of β-arabinans as the substrates for *B. aesculapii* comes from the unique genomic architecture of the extracellular β-arabinases (*hypBA2*-like genes). In this organism, one of the *hypBA2*-like extracellular β-arabinases (8B9_00889) is positioned as a downstream member of the *araQ*-*araABD* operon (Fig. 9), and very similar configurations of *araQ-araABD*-8B9_00889 *hypBA2*-like orthologues were exclusively found among species of *Bifidobacterium* isolated from gummivorous callitrichids (*B. aesculapii*, *B. primatium*, *B. parmae*, and *B. catulorum*) (Fig. S5) (29, 30, 47, 81). Coexpression of the extracellular *hypBA2*-like β-arabinase with central *araBDA* arabinose utilization genes would effectively link the expression of central arabinose metabolism to the dietary availability of β-arabinans in these species. This is quite distinct from B. longum, where genes encoding the extracellular (*hypBA2*) and intracellular (*hypBA1*) β-arabinases are part of a single operon with genes encoding a β-AOS-specific ABC-type transport system (*bauABC*) (82) that is unlinked to *araABD*. Thus, linkage between β-arabinan substrates and central arabinose metabolism in *B. aesculapii*, *B. primatium*, *B. parmae*, and *B. catulorum* may represent an evolutionary innovation that fine-tunes arabinose metabolism to the availability of a unique and common component of the host diet (β-arabinans derived from tree gums).

10.1128/mBio.01153-21.5 FIG S5 Arabinose operons from *Bifidobacterium* species isolated from callitrichids. Genomic segments of the arabinose utilization operon and *hypBA2*-like genes are aligned from genome sequences of *B. aesculapii*, *B. parmae*, *B. primatium*, and *B. catulorum*. Gene IDs are indicated for the *B. aesculapii* 8B9 genomic sequence. The *araQ* regulatory gene is colored in red, while orthologues of the *araDAB* catalytic genes are colored dark blue. The conserved *hypBA2*-like gene is colored light blue. The genomes of *B. parmae* and *B. primatium* contain genes encoding a putative Von Willebrand factor domain (vWF gene, peach color) and an exonuclease (yellow) upstream of the *hypBA2*-like orthologues, while the *B. catulorum* genome carries only the exonuclease-like gene at that position. Download FIG S5, DOCX file, 0.04 MB. Copyright © 2021 Zhu et al. 2021 Zhu et al. https://creativecommons.org/licenses/by/4.0/ This content is distributed under the terms of the Creative Commons Attribution 4.0 International license.

### Metabolic networks and predictive modeling of dietary modulation.

**FIG 10:**
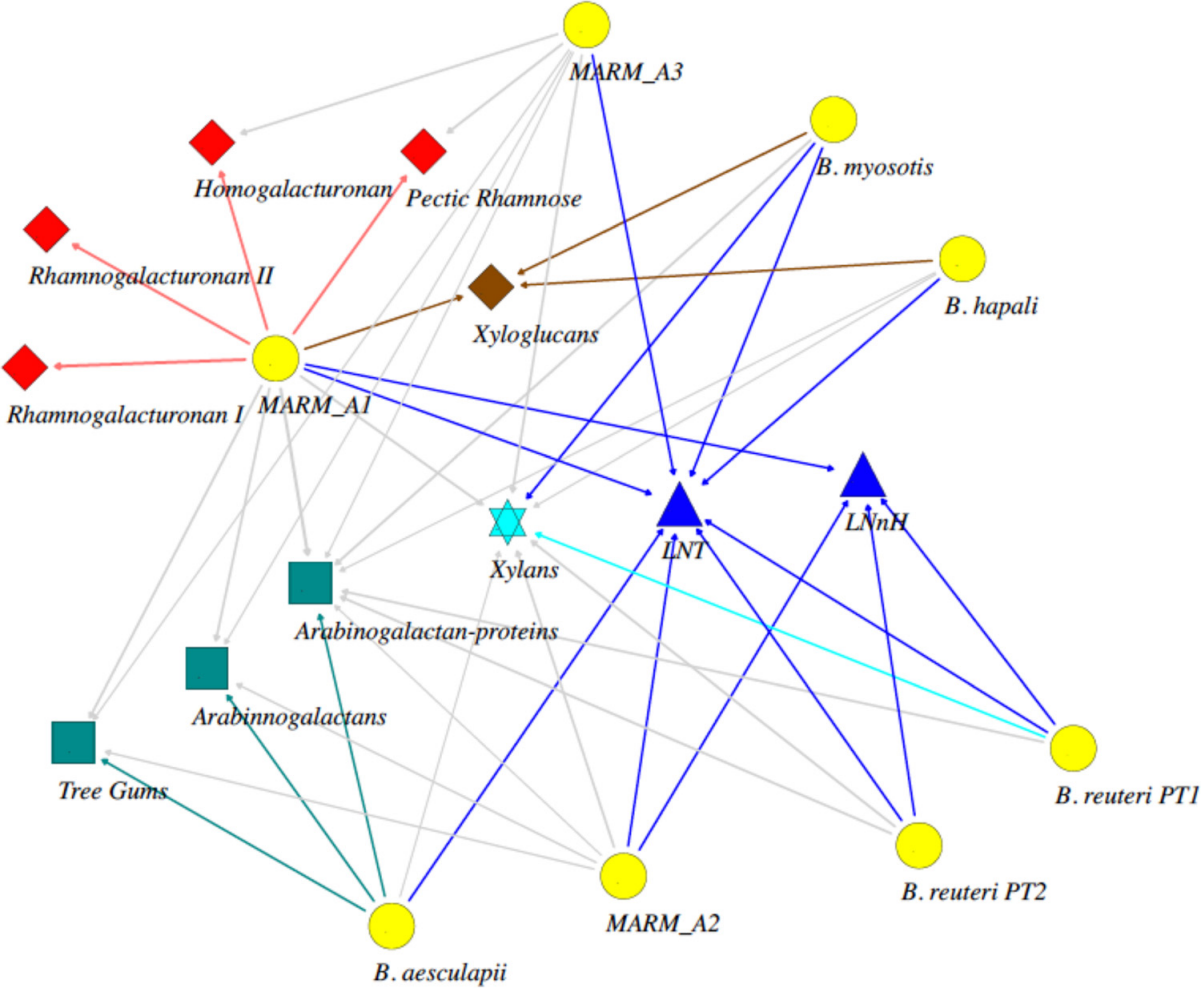
Network representation of metabolic interactions between marmoset *Bifidobacterium* species and major dietary substrates. The network was developed in SocNetV using genomic data from the individual marmoset *Bifidobacterium* species to infer capacity to serve as a primary degrader (full pathway present) or secondary cross-feeder (enzymatic capacity to degrade subcomponents) of major dietary components. Nodes representing the individual *Bifidobacterium* species and phylotypes are shown in yellow, while nodes representing major substrates are colored red (pectins), brown (xyloglucans), green (arabinogalactans), light blue (xylans), and royal blue (breastmilk oligosaccharides). Edges that are colored black indicate primary degraders and their substrates, while edges colored gray depict secondary cross-feeders. The graph was developed using a random prominence index with edges for primary degraders weighted arbitrarily at 10 and secondary degraders weighted by the number of relevant enzymes in the genome of each organism.

While much remains to be learned about the metabolic capacities and substrate specificities of the different marmoset *Bifidobacterium* species, our findings provide a framework for systematic study and predictive metabolic modeling of dietary interactions with this microbial community. The framework can be visualized through simple network analysis of the individual species, using their predicted enzyme activities to designate each organism as a primary degrader (full metabolic potential for a substrate) or a cross-feeder (metabolic capacity for degrading subcomponents of a substrate) for the major substrates in marmoset breastmilk oligosaccharide (LNT and LNnH), tree gum (AGs and AGPs), fruit (pectin and xyloglucan), and wheat bran (xylans) components of the marmoset diet (Fig. 10). Each *Bifidobacterium* species has a unique pattern of connectivity to these substrates. The two milk oligosaccharides have the highest degree of connectivity to primary degraders, whereas some of the most abundant fibers in the adult captive-marmoset diet (components of pectin and the arabinogalactan components of tree gums) have only single primary degraders. Xylans and arabinogalactan proteins also stand out uniquely as the substrates that may potentially support the entire community with one (arabinogalactan proteins) or two (xylans) primary degraders, but with each species also having the capacity to potentially cross-feed. Of course, this simple network does not include other species from the microbiome, but it does serve to illustrate how cooperative metabolic interactions can be predicted and how such predictions can be incorporated into studies of the ecological behavior of the microbiome.

## MATERIALS AND METHODS

### Sample collection.

We collected 131 fresh fecal samples from 24 captive adult common marmosets (13 females and 11 males; age range, from 2.5 to 8 years old) housed at the Callitrichid Research Center at the University of Nebraska—Omaha, NE, USA. Of the 24 marmosets, 13 (5 males and 8 females) were derived from the North Carolina pedigree (Carolina) and 11 (6 males and 5 females) were derived from the Texas pedigree (see Table S1A in the supplemental material). Marmosets were housed in enclosures as family groups or opposite-sex adult pairs. Marmosets were fed a base diet of ZuPreem (∼40%) and variable fruits (e.g., apples, melon, and bananas; ∼30%) and supplemented with hard-boiled eggs and meal worms (∼10%). Importantly, each cage also received approximately 0.5 g of gum arabic (suspended in water) to help maintain consistent dietary ingestion of tree gums. Fecal samples were collected over a 1-month period in sterilized aluminum pans, with 1 to 11 samples collected from each marmoset. Fresh fecal samples were immediately snap-frozen at −80°C.

### DNA extraction and 16 rRNA sequencing.

Total DNA was extracted using the BioSprint 96 One-For-All vet kit (Qiagen), as previously described (83). PCR amplification of the V4 region of 16S rRNA was performed using the 515F and 806R primers (84). Amplicons underwent quality control and pooling and were 2× 250-bp paired-end sequenced on the Illumina MiSeq platform at the Nebraska Food for Health Center at the University of Nebraska—Lincoln.

### 16S rRNA analysis.

We examined our 16S rRNA gut microbiome data from common marmosets generated in this study in addition to raw 16S rRNA V4 region data set 25 for other primate species. From animals sampled at the CRC, we obtained 6,672,027 high-quality reads, ranging from 13,951 to 109,610 reads per sample. For comparison across primate species, data from this study was combined with 1,287 fecal samples from 25 primate species derived from the Primate Microbiome Project, including nine hominids, 17 Old World monkeys, six New World monkeys, and three prosimians (Table S1C). All data underwent quality control by trimming and deletion of chimeras, which resulted in a cleaned data set (length, ∼250 bp) of 13,000 reads (normalized) per sample. Sample reads were preclustered into OTUs based on 97% similarity with USEARCH sequences, and singletons were removed (85). Representative sequences of each OTU were obtained, and reads were assigned using the USEARCH algorithm (*de novo* OTU picking). Taxonomic annotation was assigned with UCLUST using a similarity of 80% against the SILVA 16S rRNA gene database. The final OTU table with taxonomy information was obtained using QIIME 2 (86), and this table was subsequently used for all downstream analyses. The Bray-Curtis distance based on a genus abundance of 1,418 fecal microbiota was used for hierarchical clustering analysis via Vegan and Ape (87–90). In addition, the partitioning around medoids (PAM) clustering algorithm was also used for clustering analysis based on genus abundance (91). The ComplexHeatmap package was used to plot high-abundance genera in 1,418 fecal samples (92). All analyses using packages were done in RStudio (93). Tree files for hierarchical clustering analysis were visualized in FigTree (94). The Mann-Whitney test was performed to detect significant differences between origins and sexes.

### *Bifidobacterium* isolation from marmoset fecal samples from seven individuals.

*Bifidobacterium* isolates were obtained from adult marmoset fecal samples (*n* = 7) that had a high abundance of *Bifidobacterium* spp. A portion (0.1 g) of each fecal sample was weighed out, and 10-fold serial dilutions with sterile phosphate-buffered saline (PBS) were carried out. Dilutions were plated onto MRS (De Man, Rogosa, and Sharpe) medium and BSIM (*Bifidobacterium* selective iodoacetate mupirocin) (95) agar plates. The plates were incubated anaerobically at 37°C for 48 h, and 10 colonies were picked from each plate into prereduced MRS broth. Plates were incubated anaerobically at 37°C for 24 h. One milliliter of each culture was used for subsequent DNA extraction as previously described (96). Universal 16S rRNA bacterial primers 27F (5′-AGAGTTTGATCCTGGCTCAG-3′) and 1392R (5′-GGTTACCTTGTTACGACTT-3′) were used to amplify the bacterial 16S rRNA gene. PCR products were visualized on a 1% agarose gel stained with ethidium bromide under UV light to confirm the presence of an ∼1,350-bp band. PCR products were purified using the QIAquick PCR purification kit (Qiagen, USA) prior to bidirectional Sanger sequencing at the Michigan State University RTSF Genomics Core. DNAStar was used to assemble the forward and reverse sequences from 116 isolates. A BLAST search against the National Center for Biotechnology Information (NCBI) database (97) showed that 72 isolates had ≥89% identity to one or more species of *Bifidobacterium* (Table S2A). From this, we chose a diverse set of 18 *Bifidobacterium* isolates for the subsequent whole-genome sequencing based on their 16S rRNA taxonomic assignment and characteristics of the host.

The 18 *Bifidobacterium* isolates were subcultured in prereduced MRS broth and incubated anaerobically for 24 to 48 h at 37°C. To ensure that the cultures were pure, each inoculum was streaked onto MRS medium plates, and single colonies were picked and reinoculated into MRS broth. Inoculums from the 18 isolates were used for subsequent DNA extraction by a PureLink Microbiome DNA purification kit (Invitrogen). Whole-genome DNA library construction was prepared according to the protocol from Illumina’s Nextera XT DNA Library Prep kit. Whole-genome sequencing was performed on the Illumina MiSeq sequencer at the Nebraska Food for Health Center at the University of Nebraska—Lincoln.

### CAZy and KEGG prediction.

The raw reads of each strain were assembled by SPAdes 4.01 (98). The *N*_50_ for each draft genome was at a reasonable level (Tables S2B and S2C). The coverage of each draft genome was calculated with Salmon (99). Glimmer (v3.02) was used to predict the putative genes in the 18 marmoset-originating *Bifidobacterium* strains described here and 20 other published *Bifidobacterium* genomes of human origin (100). The carbohydrate-active enzymes in each genome were predicted using HMMER (v3.1b2) and CAZy (v6) (101, 102). The KEGG metabolism pathway was predicted by BLAST (v2.2.35) and KEGG (v59) (103). CAZy profiles (glycoside hydrolases [GHs], glycosyltransferases [GTs], carbohydrate-binding modules [CBMs], PLs, carbohydrate esterases [CEs], and auxiliary activities [AAs]) were used to construct a hierarchical clustering of the 38 above-described *Bifidobacterium* genomes with heatmap.2 in RStudio (104). The significantly differential abundant feature detections of KEGG pathways among groups were used in LEfSe (linear discriminant analysis effect size) (105).

### Phylogenetic reconstructions.

We downloaded published genomes from 64 isolates comprising 45 *Bifidobacterium* species from the NCBI for comparative genomic analysis (Table S2E). dbCAN was used to annotate GH profiles for each genome (106). Finally, the entire data set of 64 *Bifidobacterium* genomes plus genomes from the 18 marmoset isolates were used for core gene analysis (Table S2B). The genomes were annotated by DFAST (107), and their putative KEGG profiles and clusters of orthologous groups (COG) were generated using BGPA (108). The neighbor-joining phylogeny was reconstructed using concatenated core gene sequences by MEGA7 with 500 bootstrap replications (109). The linear interactive genome visualization was generated by DNAPlotter (110).

### qPCR analysis of total *Bifidobacterium* and individual taxa of marmoset *Bifidobacterium* species.

To quantify the different species/taxa of *Bifidobacterium* isolated from the marmosets in our study, we developed a set of qPCR primers to detect relevant taxonomic units represented by these isolates. Phylogeny was used as the basis for identifying taxonomic units, with candidate genes being identified at the levels of genus, species, and phylotype (subtypes within a species). The genomes of each of the 18 marmoset *Bifidobacterium* genomes were first assembled with Spades (98), and the assemblies were annotated with Prokka (111) on Amazon Web Services (AWS) so that assemblies of different *Bifidobacterium* species could be used as reference genomes. The list of *Bifidobacterium* genome assemblies used can be found on GitHub (https://github.com/jcgneto/bifidobacterium/blob/master/list_reference_bifido_genomes.txt) along with instructions on how to install Prokka on AWS (https://github.com/jcgneto/installing_conda_and_prokka_aws).

Phylogeny was inferred from core genome alignments developed in FastTree (112) using a generalized time reversible (GTR) model (113) and subsequently visualized in Phandango (114). As with the core genome-based phylogeny of our isolates in Fig. 2, this phylogenetic analysis detected 7 major taxonomic groups among the 18 genomes, corresponding to the different species (*B. reuteri*, *B. myosotis*, *B. aesculapii*, *B. hapali*, MARM_A1, MARM_A2, and MARM_A3). Using the core genome phylogenies of these species, we next determined if the pan-genomic content of each species could define significant subtypes (phylotypes) that would be relevant for quantification by qPCR. This was accomplished with an SNP-sites analysis (115) of the .aln output files from Roary and filtering putative recombination regions with Gubbins (116). The FastTree-based phylogeny produced by Gubbins was visualized in Phandango along with the gene presence/absence output from Roary. This analysis detected the expected significant differences in pan-genomic content between each species and also showed that two distinct phylotypes of *B. reuteri* (phylotype 1 and phylotype 2) were represented among the isolates.

Based on the pan-genomic analyses at the species and phylotype (*B. reuteri*) levels, candidate genes were chosen for development of qPCR primers to detect each of the different species (*B. aesculapii*, *B. myosotis*, *B. hapali*, MARM_A1, MARM_A2, and MARM_A3) and the two major phylotypes of *B. reuteri* (*B. reuteri* phylotype 1, *B. reuteri* phylotype 2). Criteria for prioritizing candidate genes were based on (i) the uniqueness of the gene to the taxon among the 18 marmoset isolates, (ii) the uniqueness of the candidate genes from core genomic content across multiple *Bifidobacterium* genomes, and (iii) the longest unique genomic segments. The Roary scripts used to define candidates based on these criteria are indicated below.

1. unique_phylogroup1.sh (get a list of phylogroup1 unique genes)
2. unique_phylogroup2.sh (get a list of phylogroup2 unique genes)
3. unique_phylogroup3.sh (get a list of phylogroup3 unique genes)
4. unique_phylogroup4.sh (get a list of phylogroup4 unique genes)
5. unique_phylogroup5.sh (get a list of phylogroup5 unique genes)
6. phylogroup_1_phylotype_1.sh (get a list of phylogroup_1_phylotype_1 unique genes)
7. phylogroup_1_phylotype_2.sh (get a list of phylogroup_1_phylotype_2 unique genes)
8. phylogroup_4_phylotype_1.sh (get a list of phylogroup_4_phylotype_1 unique genes)
9. phylogroup_4_phylotype_2.sh (get a list of phylogroup_4_phylotype_2 unique genes)
10. phylogroup_4_phylotype_3.sh (get a list of phylogroup_4_phylotype_3 unique genes)

From the list of prioritized candidate genes for each taxon, the rapid identification of PCR primers for unique core sequences (RUCS) program was used to design primers (117). Primer specificity was confirmed first by using FastPCR against the DNA from all 18 isolates (118) and subsequently by a BLAST search against the NCBI RefSeq representative genome database for bacteria with NCBI Primer BLAST. The optimum annealing temperature of the candidate qPCR primers was determined empirically by thermal-gradient PCR, which showed that 60°C provided the most sensitivity and specificity across the combined set of qPCR primers. The primers are listed in Table S2D.

All qPCR assays were performed with an Applied Biosystems QuantStudio 5 real-time 384-well PCR system (Thermo Fisher Scientific, MA, USA) using a 60°C melting temperature. Each reaction mixture contained 10 μl of the qPCR master mix (2× Maxima SYBR green; Thermo Fisher Scientific, MA, USA), 0.25 μM specific primers for each species or phylotype, 8 μl of water, and 1 μl of the template DNA, for a final volume of 20 μl. Each DNA sample was tested in duplicate reactions. For each target, standard curves were made using a series of 10-fold serial dilutions of DNA isolated from pure cultures whose cells had been enumerated by plate counting. Each qPCR primer pair was validated against isolates of nontarget marmoset-derived *Bifidobacterium* species (for species-specific and subtype-specific primers) as well as individual isolates of human-derived *Bifidobacterium* species (B. longum, *B. adolescentis*, *B. animalis*, *B. pseudocatenulatum*). Data analysis used LOWESS (Locally Weighted Scatterplot Smoothing) (119) to identify the trend of either the absolute abundance of *Bifidobacterium* organisms or the relative proportion of each phylogroup and phylotype by the number of days of age of the marmoset.

### Carbohydrate fermentation.

Growth curves were generated by a 1:100 dilution of overnight anaerobic cultures of each strain in MRS medium into 20 ml of fresh basal MRS medium (bMRS; MRS medium devoid of glucose) containing 1% glucose, 1% gum arabic, or no added carbohydrate, and the cultures were incubated anaerobically at 37°C. The OD at 600 nm (OD_600_) measurements were taken from samples at the 0-, 4-, 8-, 12-, and 24-h time points after inoculation. In a second experiment, growth curves were generated with a 1:100 dilution of overnight anaerobic cultures of each strain in MRS medium into microcultures in 96-well plates (200-μl volumes) containing bMRS plus 1% glucose, arabinose, galactose, larch wood arabinogalactan, or no added carbohydrate. The cultures were inoculated in triplicate for each strain in 96-well plates and were incubated in a plate reader (Sunrise; Tecan, Switzerland) anaerobically at 37°C. OD_600_ values were measured in each well every 20 min over a period of 48 h (approximately 150 measurements).

### Ethics statement.

All animal procedures were reviewed and approved by the University of Nebraska Medical Center/University of Nebraska at Omaha Institutional Animal Care and Use Committee (IACUC no. 16-104) and adhered to the American Society of Primatologists' Principles for the Ethical Treatment of Non-Human Primates (https://www.asp.org/2021/04/20/principles-for-the-ethical-treatment-of-non-human-primates/).

### Data availability.

The raw 16S amplicon and whole-genome sequencing data can be found in the NCBI database under accession numbers PRJNA679696 and PRJNA679695, respectively.
