# Supplementary material for: Captive Common Marmosets (Callithrix jacchus) Are Colonized throughout Their Lives by a Community of Bifidobacterium Species with Species-Specific Genomic Content That Can Support Adaptation to Distinct Metabolic Niches
Source: mBio. 2021 Aug 3;12(4):e01153-21. doi: 10.1128/mBio.01153-21 (PMC8406136; doi:10.1128/mBio.01153-21)
Supplement: TABLE S1 [file mbio.01153-21-st001.docx]

**Table S1 The sample information in this study.**

**Table S1A.** Detailed information of the 131 common marmoset fecal samples collected from 24 common marmosets.

| Sample | Animal | Sex | Origin | Collection Date |
| --- | --- | --- | --- | --- |
| MM6 | Abu | M | Carolina | 11/08/2016 |
| MM13 | Abu | M | Carolina | 11/10/2016 |
| MM27 | Abu | M | Carolina | 11/14/2016 |
| MM39 | Abu | M | Carolina | 11/16/2016 |
| MM46 | Abu | M | Carolina | 11/17/2016 |
| MM74 | Abu | M | Carolina | 11/21/2016 |
| MM85 | Abu | M | Carolina | 11/22/2016 |
| MM99 | Abu | M | Carolina | 11/24/2016 |
| MM124 | Abu | M | Carolina | 11/28/2016 |
| MM159 | Abu | M | Carolina | 12/05/2016 |
| MM4 | Artemis | F | Carolina | 11/08/2016 |
| MM18 | Artemis | F | Carolina | 11/10/2016 |
| MM28 | Artemis | F | Carolina | 11/14/2016 |
| MM36 | Artemis | F | Carolina | 11/16/2016 |
| MM51 | Artemis | F | Carolina | 11/17/2016 |
| MM80 | Artemis | F | Carolina | 11/21/2016 |
| MM123 | Artemis | F | Carolina | 11/28/2016 |
| MM135 | Artemis | F | Carolina | 11/29/2016 |
| MM148 | Arwen | F | Texas | 12/02/2016 |
| MM24 | Athena | F | Carolina | 11/12/2016 |
| MM30 | Athena | F | Carolina | 11/14/2016 |
| MM33 | Athena | F | Carolina | 11/15/2016 |
| MM64 | Athena | F | Carolina | 11/18/2016 |
| MM68 | Athena | F | Carolina | 11/19/2016 |
| MM72 | Athena | F | Carolina | 11/20/2016 |
| MM83 | Athena | F | Carolina | 11/21/2016 |
| MM97 | Athena | F | Carolina | 11/23/2016 |
| MM120 | Athena | F | Carolina | 11/26/2016 |
| MM121 | Athena | F | Carolina | 11/28/2016 |
| MM156 | Athena | F | Carolina | 12/05/2016 |
| MM15 | Batman | M | Carolina | 11/10/2016 |
| MM25 | Batman | M | Carolina | 11/13/2016 |
| MM43 | Batman | M | Carolina | 11/16/2016 |
| MM48 | Batman | M | Carolina | 11/17/2016 |
| MM65 | Batman | M | Carolina | 11/19/2016 |
| MM69 | Batman | M | Carolina | 11/20/2016 |
| MM77 | Batman | M | Carolina | 11/21/2016 |
| MM101 | Batman | M | Carolina | 11/24/2016 |
| MM115 | Batman | M | Carolina | 11/26/2016 |
| MM45 | Bello | M | Texas | 11/16/2016 |
| MM55 | Bello | M | Texas | 11/17/2016 |
| MM109 | Bello | M | Texas | 11/25/2016 |
| MM61 | Cletus | M | Texas | 11/18/2016 |
| MM5 | Dacron | M | Carolina | 11/08/2016 |
| MM17 | Dacron | M | Carolina | 11/10/2016 |
| MM29 | Dacron | M | Carolina | 11/14/2016 |
| MM41 | Dacron | M | Carolina | 11/16/2016 |
| MM50 | Dacron | M | Carolina | 11/17/2016 |
| MM79 | Dacron | M | Carolina | 11/21/2016 |
| MM94 | Dacron | M | Carolina | 11/23/2016 |
| MM134 | Dacron | M | Carolina | 11/29/2016 |
| MM137 | Dexter | M | Texas | 11/29/2016 |
| MM12 | Ellie | F | Carolina | 11/09/2016 |
| MM16 | Ellie | F | Carolina | 11/10/2016 |
| MM26 | Ellie | F | Carolina | 11/13/2016 |
| MM42 | Ellie | F | Carolina | 11/16/2016 |
| MM49 | Ellie | F | Carolina | 11/17/2016 |
| MM66 | Ellie | F | Carolina | 11/19/2016 |
| MM70 | Ellie | F | Carolina | 11/20/2016 |
| MM78 | Ellie | F | Carolina | 11/21/2016 |
| MM90 | Ellie | F | Carolina | 11/23/2016 |
| MM102 | Ellie | F | Carolina | 11/24/2016 |
| MM145 | Ellie | F | Carolina | 12/02/2016 |
| MM3 | Fabio | M | Carolina | 11/08/2016 |
| MM23 | Fabio | M | Carolina | 11/12/2016 |
| MM34 | Fabio | M | Carolina | 11/15/2016 |
| MM67 | Fabio | M | Carolina | 11/19/2016 |
| MM71 | Fabio | M | Carolina | 11/20/2016 |
| MM84 | Fabio | M | Carolina | 11/21/2016 |
| MM98 | Fabio | M | Carolina | 11/23/2016 |
| MM108 | Fabio | M | Carolina | 11/25/2016 |
| MM122 | Fabio | M | Carolina | 11/28/2016 |
| MM157 | Fabio | M | Carolina | 12/05/2016 |
| MM19 | Harlow | F | Carolina | 11/10/2016 |
| MM56 | Harlow | F | Carolina | 11/18/2016 |
| MM132 | Harlow | F | Carolina | 11/28/2016 |
| MM160 | Harlow | F | Carolina | 12/05/2016 |
| MM59 | Izla | F | Carolina | 11/18/2016 |
| MM166 | Izla | F | Carolina | 12/07/2016 |
| MM21 | Jax | M | Carolina | 11/12/2016 |
| MM31 | Jax | M | Carolina | 11/14/2016 |
| MM52 | Jax | M | Carolina | 11/17/2016 |
| MM63 | Jax | M | Carolina | 11/18/2016 |
| MM81 | Jax | M | Carolina | 11/21/2016 |
| MM96 | Jax | M | Carolina | 11/23/2016 |
| MM106 | Jax | M | Carolina | 11/25/2016 |
| MM133 | Jax | M | Carolina | 11/29/2016 |
| MM88 | Khaleesi | F | Texas | 11/22/2016 |
| MM147 | Khaleesi | F | Texas | 12/02/2016 |
| MM37 | Liilu | F | Carolina | 11/16/2016 |
| MM75 | Liilu | F | Carolina | 11/21/2016 |
| MM91 | Liilu | F | Carolina | 11/23/2016 |
| MM114 | Liilu | F | Carolina | 11/25/2016 |
| MM44 | Lily | F | Texas | 11/16/2016 |
| MM58 | Lily | F | Texas | 11/18/2016 |
| MM87 | Lily | F | Texas | 11/22/2016 |
| MM110 | Lily | F | Texas | 11/25/2016 |
| MM130 | Lily | F | Texas | 11/28/2016 |
| MM144 | Lily | F | Texas | 12/01/2016 |
| MM161 | Lily | F | Texas | 12/05/2016 |
| MM2 | Nikko | M | Texas | 11/03/2016 |
| MM60 | Nikko | M | Texas | 11/18/2016 |
| MM112 | Nylon | F | Carolina | 11/25/2016 |
| MM146 | Nylon | F | Carolina | 12/02/2016 |
| MM125 | Odie | F | Texas | 11/28/2016 |
| MM136 | Odie | F | Texas | 11/29/2016 |
| MM142 | Odie | F | Texas | 12/01/2016 |
| MM38 | Tank | M | Texas | 11/16/2016 |
| MM76 | Tank | M | Texas | 11/21/2016 |
| MM113 | Tank | M | Texas | 11/25/2016 |
| MM149 | Tank | M | Texas | 12/05/2016 |
| MM20 | Twix | M | Texas | 11/10/2016 |
| MM54 | Twix | M | Texas | 11/17/2016 |
| MM57 | Twix | M | Texas | 11/18/2016 |
| MM111 | Twix | M | Texas | 11/25/2016 |
| MM14 | Ursa | F | Texas | 11/10/2016 |
| MM32 | Ursa | F | Texas | 11/14/2016 |
| MM40 | Ursa | F | Texas | 11/16/2016 |
| MM47 | Ursa | F | Texas | 11/17/2016 |
| MM73 | Ursa | F | Texas | 11/21/2016 |
| MM89 | Ursa | F | Texas | 11/23/2016 |
| MM100 | Ursa | F | Texas | 11/24/2016 |
| MM158 | Ursa | F | Texas | 12/05/2016 |
| MM7 | Velcro | F | Carolina | 11/08/2016 |
| MM22 | Velcro | F | Carolina | 11/12/2016 |
| MM35 | Velcro | F | Carolina | 11/16/2016 |
| MM53 | Velcro | F | Carolina | 11/17/2016 |
| MM62 | Velcro | F | Carolina | 11/18/2016 |
| MM82 | Velcro | F | Carolina | 11/21/2016 |
| MM118 | Velcro | F | Carolina | 11/26/2016 |
| MM154 | Velcro | F | Carolina | 12/05/2016 |

**Table S1B.** The dominant genera in the gut microbiome of 131 common marmoset fecal samples in this study.

| **SampleID** | ***Bifidobacterium*** | ***Bacteroides*** | ***Collinsella*** | ***Prevotella 9*** | ***Phascolarctobacterium*** | ***Megamonas*** | ***Megasphaera*** | ***Escherichia-Shigella*** | ***Alloprevotella*** | ***Parabacteroides*** | ***Olsenella*** | ***Syntrophococcus*** |
| --- | --- | --- | --- | --- | --- | --- | --- | --- | --- | --- | --- | --- |
| MM27 | 0.275 | 0.068462 | 0.103077 | 0.001923 | 0.040846 | 0.187385 | 0.059077 | 0.087385 | 0.055 | 0.003308 | 0.015846 | 0.020846 |
| MM74 | 0.208 | 0.150231 | 0.163615 | 0.001077 | 0.066385 | 0.060846 | 0.038923 | 0.010385 | 0.000308 | 0.013385 | 0.026692 | 0.017462 |
| MM99 | 0.291 | 0.216615 | 0.058846 | 0.023385 | 0.078769 | 0.061462 | 0.059692 | 0.028 | 0.016 | 0.022538 | 0.019538 | 0.016 |
| MM159 | 0.345308 | 0.069846 | 0.030077 | 0.208538 | 0.076538 | 0.008 | 0.063538 | 0.014462 | 0.047538 | 0.018462 | 0.007923 | 0.008308 |
| MM6 | 0.192846 | 0.162615 | 0.078308 | 0.216077 | 0.060385 | 0.021308 | 0.019077 | 0.053385 | 0.000692 | 0.025 | 0.023538 | 0.012462 |
| MM13 | 0.207385 | 0.118 | 0.101769 | 0.207538 | 0.099462 | 0.019538 | 0 | 0.038923 | 0 | 0.016385 | 0.020692 | 0.017846 |
| MM39 | 0.262846 | 0.124154 | 0.105231 | 0.197 | 0.052692 | 0.022077 | 0.001154 | 0.027154 | 0.007769 | 0.010615 | 0.031615 | 0.025923 |
| MM46 | 0.228154 | 0.090923 | 0.092769 | 0.214308 | 0.137385 | 0.028769 | 0.000308 | 0.000154 | 7.69E-05 | 0.018 | 0.011 | 0.019615 |
| MM85 | 0.258231 | 0.270923 | 0.085231 | 0.006692 | 0.029 | 0.000308 | 0.047846 | 0.082692 | 0.000231 | 0.036077 | 0.021692 | 0.017462 |
| MM124 | 0.309615 | 0.186308 | 0.045462 | 0.092615 | 0.022923 | 0.024077 | 0.000462 | 0.102692 | 0 | 0.022615 | 0.054462 | 0.021231 |
| MM4 | 0.318385 | 0.090692 | 0.073077 | 0.020231 | 0.024231 | 0.079846 | 0.126231 | 0.028154 | 0.057385 | 0.007615 | 0.009154 | 0.009692 |
| MM36 | 0.262 | 0.225 | 0.069615 | 0.004615 | 0.047077 | 0.079538 | 0.036692 | 0.019308 | 0.106308 | 0.011538 | 0.013923 | 0.018154 |
| MM80 | 0.167692 | 0.264 | 0.160769 | 0.000231 | 0.024385 | 0.021462 | 0.018231 | 0 | 0.074154 | 0.015462 | 0.019154 | 0.018769 |
| MM18 | 0.326308 | 0.155231 | 0.122385 | 0.022692 | 0.112 | 0.000692 | 0.001 | 0.038 | 0.001538 | 0.012692 | 0.014385 | 0.043538 |
| MM28 | 0.205154 | 0.107385 | 0.088923 | 0.215846 | 0.109846 | 0.030846 | 0.000538 | 0.018769 | 0.000154 | 0.021923 | 0.018923 | 0.029154 |
| MM51 | 0.226769 | 0.221077 | 0.125923 | 0.001154 | 0.062308 | 7.69E-05 | 0.071769 | 0.000462 | 0 | 0.094154 | 0.032769 | 0.026538 |
| MM123 | 0.208692 | 0.278231 | 0.092769 | 0.000923 | 0.035692 | 0.000385 | 0.030769 | 0.008 | 0.000231 | 0.102615 | 0.069538 | 0.033231 |
| MM135 | 0.265923 | 0.217077 | 0.089923 | 0.038615 | 0.071846 | 0.000385 | 0.000923 | 0.143077 | 0.000308 | 0.036385 | 0.019846 | 0.017462 |
| MM148 | 0.250077 | 0.148385 | 0.065231 | 0.145308 | 0.050462 | 0.020308 | 0.003 | 0.053 | 0.000154 | 0.028923 | 0.042462 | 0.020692 |
| MM33 | 0.280231 | 0.182 | 0.115077 | 0.003538 | 0.042462 | 0.031385 | 0.051846 | 0.062615 | 0.065385 | 0.003923 | 0.005923 | 0.013385 |
| MM64 | 0.265 | 0.137077 | 0.087769 | 0.000692 | 0.045462 | 0.047154 | 0.017615 | 0.000154 | 0.143846 | 0.006462 | 0.010923 | 0.012846 |
| MM68 | 0.449769 | 0.030846 | 0.049769 | 0.000154 | 0.011462 | 0.125462 | 0.168077 | 0.000154 | 0.106538 | 0.011846 | 0.002231 | 0.000154 |
| MM72 | 0.168385 | 0.188 | 0.053 | 0.000308 | 0.180692 | 0.046692 | 0.024308 | 0.013923 | 0.124846 | 0.024077 | 0.015615 | 0.014231 |
| MM83 | 0.321846 | 0.251615 | 0.082923 | 0.013615 | 0.045462 | 0.023385 | 0.032385 | 0.086615 | 7.69E-05 | 0.021923 | 0.012308 | 0.009231 |
| MM156 | 0.321385 | 0.193077 | 0.068692 | 0.000385 | 0.051077 | 0.158077 | 0.028692 | 0.006077 | 0.008 | 0.013769 | 0.005385 | 0.006538 |
| MM24 | 0.263231 | 0.220385 | 0.118538 | 0.024692 | 0.062385 | 0.000538 | 0.000462 | 0.040385 | 0.000231 | 0.019846 | 0.017923 | 0.025615 |
| MM30 | 0.255462 | 0.029692 | 0.046538 | 0.337231 | 0.210385 | 0.000615 | 0.000154 | 0.006846 | 0.002462 | 0.007769 | 0.003077 | 0.010385 |
| MM97 | 0.288846 | 0.185615 | 0.087385 | 0.041538 | 0.041923 | 0.001154 | 0.027923 | 0.073 | 0 | 0.032 | 0.023154 | 0.020692 |
| MM120 | 0.186692 | 0.230077 | 0.047462 | 0.036769 | 0.119846 | 0.010615 | 0 | 0.064769 | 0.000231 | 0.029615 | 0.029538 | 0.021538 |
| MM121 | 0.298462 | 0.227308 | 0.102154 | 0.014385 | 0.037538 | 0 | 7.69E-05 | 0.109231 | 7.69E-05 | 0.050692 | 0.031615 | 0.021 |
| MM48 | 0.243154 | 0.152462 | 0.049077 | 0.004385 | 0.103 | 0.038077 | 0.064769 | 0.129 | 0.120154 | 0.006462 | 0.004692 | 0.008692 |
| MM65 | 0.131308 | 0.251462 | 0.057769 | 0.014923 | 0.100538 | 0.038692 | 0.021692 | 7.69E-05 | 0.199231 | 0.011846 | 0.007923 | 0.012769 |
| MM69 | 0.180923 | 0.283923 | 0.069538 | 0.001923 | 0.074154 | 0.112538 | 0.069077 | 0.001462 | 0.059462 | 0.012846 | 0.007615 | 0.013538 |
| MM77 | 0.094769 | 0.356308 | 0.091769 | 0.000462 | 0.046692 | 0.027 | 0.006923 | 0.000231 | 0.148385 | 0.035231 | 0.017385 | 0.016231 |
| MM101 | 0.334154 | 0.130538 | 0.082846 | 0.050923 | 0.049308 | 0.023615 | 0.019692 | 0.010923 | 0.045 | 0.015308 | 0.009923 | 0.018077 |
| MM15 | 0.221692 | 0.090231 | 0.024692 | 0.051769 | 0.033769 | 0 | 0.000308 | 0.138231 | 0 | 0.011846 | 0.003538 | 0.010769 |
| MM25 | 0.298077 | 0.139 | 0.082462 | 0.172308 | 0.065154 | 0.000154 | 0.000154 | 0.038231 | 0.000154 | 0.031308 | 0.009154 | 0.029846 |
| MM43 | 0.289923 | 0.080538 | 0.062385 | 0.134769 | 0.11 | 0.055538 | 0.000385 | 0.000154 | 7.69E-05 | 0.007 | 0.014154 | 0.031077 |
| MM115 | 0.359923 | 0.098538 | 0.103923 | 0.098692 | 0.091462 | 0.000231 | 0.000615 | 0.017231 | 0 | 0.036154 | 0.018538 | 0.027846 |
| MM45 | 0.476385 | 0.007692 | 0.198846 | 0.000769 | 0.015231 | 0.025 | 0.087462 | 0.016615 | 0.004308 | 0.000846 | 0.013308 | 0.039692 |
| MM55 | 0.159308 | 0.162308 | 0.031538 | 0.048692 | 0.070538 | 0.168769 | 0.029154 | 0.005923 | 0.091 | 0.015154 | 0.002462 | 0.001769 |
| MM109 | 0.216846 | 0.132615 | 0.047615 | 0.270538 | 0.062154 | 0.038615 | 0.000615 | 0.006 | 0 | 0.026769 | 0.008308 | 0.016462 |
| MM61 | 0.379692 | 0.052077 | 0.058923 | 0.000385 | 0.054923 | 0.142308 | 0.221154 | 0.000769 | 0.000846 | 0.000923 | 0.006769 | 0.011462 |
| MM5 | 0.354231 | 0.234308 | 0.064769 | 0.002692 | 0.027231 | 0.037 | 0.04 | 0.004462 | 0.083538 | 0.020077 | 0.008846 | 0.016385 |
| MM29 | 0.125769 | 0.015769 | 0.533308 | 0.000538 | 0.010308 | 0.015231 | 0.026231 | 0.088846 | 0 | 0.001615 | 0.022462 | 0.042 |
| MM94 | 0.303154 | 0.175462 | 0.073231 | 0.052231 | 0.035 | 0.057923 | 0.026846 | 0.060615 | 0.000615 | 0.021538 | 0.037 | 0.022385 |
| MM17 | 0.132692 | 0.133385 | 0.085615 | 0.246308 | 0.103538 | 0.034077 | 0.000308 | 0.032769 | 0.000923 | 0.024385 | 0.022 | 0.012308 |
| MM41 | 0.249308 | 0.116385 | 0.108538 | 0.211 | 0.129077 | 0.024538 | 0.000385 | 0.000462 | 0.000462 | 0.010769 | 0.014923 | 0.024692 |
| MM50 | 0.258077 | 0.104846 | 0.134385 | 0.226154 | 0.089615 | 0.034308 | 0.000231 | 0.000308 | 0.000231 | 0.014385 | 0.008769 | 0.019308 |
| MM79 | 0.310923 | 0.181231 | 0.135231 | 0.002385 | 0.026154 | 7.69E-05 | 0.061615 | 0.127231 | 0.000308 | 0.033462 | 0.020692 | 0.015385 |
| MM134 | 0.256538 | 0.151846 | 0.055 | 0.261769 | 0.095154 | 7.69E-05 | 7.69E-05 | 0.019769 | 0 | 0.033308 | 0.009538 | 0.015385 |
| MM137 | 0.640308 | 0.010769 | 0.107308 | 0.004 | 0.005231 | 0.015385 | 0 | 0.008077 | 0 | 0.002 | 0.016308 | 0.039308 |
| MM26 | 0.253692 | 0.172385 | 0.091154 | 0.007769 | 0.036231 | 0.093462 | 0.072 | 0.017538 | 0.080308 | 0.013385 | 0.019308 | 0.022538 |
| MM42 | 0.277615 | 0.178923 | 0.090769 | 0.005769 | 0.050923 | 0.137231 | 0.048 | 0.011462 | 0.080538 | 0.009308 | 0.014692 | 0.020692 |
| MM49 | 0.138385 | 0.025154 | 0.023538 | 0.257923 | 0.205462 | 0.011615 | 0.031154 | 0.000308 | 0.117077 | 0.019385 | 0.011154 | 0.006538 |
| MM66 | 0.181077 | 0.226538 | 0.193538 | 0.000231 | 0.051538 | 0.041231 | 0.050615 | 0.010231 | 0 | 0.021615 | 0.033308 | 0.017769 |
| MM70 | 0.250154 | 0.116 | 0.054923 | 0.056385 | 0.083308 | 0.067538 | 0.070308 | 0.021231 | 0.198923 | 0.008462 | 0.003923 | 0.004692 |
| MM90 | 0.288615 | 0.176692 | 0.070231 | 0.047538 | 0.036846 | 0.036154 | 0.033846 | 0.008692 | 0.086077 | 0.028615 | 0.028769 | 0.020077 |
| MM145 | 0.223 | 0.223 | 0.130385 | 0.000308 | 0.021615 | 0.021846 | 0.027 | 0.103692 | 0.000462 | 0.021615 | 0.034615 | 0.015923 |
| MM12 | 0.175923 | 0.232385 | 0.088615 | 0.014538 | 0.047615 | 0.000231 | 0.000231 | 0.250154 | 0.000154 | 0.046 | 0.016615 | 0.020769 |
| MM16 | 0.162 | 0.127231 | 0.047923 | 0.371923 | 0.087615 | 0 | 7.69E-05 | 0.008923 | 0.000154 | 0.021769 | 0.007846 | 0.021615 |
| MM78 | 0.24 | 0.112 | 0.049769 | 0.266154 | 0.074154 | 0.034231 | 0.000154 | 0.054846 | 0.002462 | 0.010385 | 0.032462 | 0.019308 |
| MM102 | 0.264923 | 0.115 | 0.042231 | 0.262154 | 0.053462 | 0.048385 | 0.002923 | 0.007769 | 0.005462 | 0.015846 | 0.043308 | 0.029692 |
| MM23 | 0.373923 | 0.093077 | 0.244462 | 0.001769 | 0.031077 | 0.040923 | 0.063692 | 0.003923 | 0.061154 | 0.008 | 0.005308 | 0.008538 |
| MM71 | 0.098231 | 0.093231 | 0.111615 | 0.000231 | 0.149231 | 0.042462 | 0.036231 | 0 | 0.162077 | 0.017154 | 0.014846 | 0.018154 |
| MM98 | 0.316538 | 0.160846 | 0.063692 | 0.045692 | 0.062231 | 0.037769 | 0.045615 | 0.060923 | 0.030308 | 0.015 | 0.026769 | 0.013769 |
| MM108 | 0.273692 | 0.204615 | 0.091692 | 0.007 | 0.046077 | 0.103769 | 0.060923 | 0.036769 | 0 | 0.020077 | 0.008385 | 0.009692 |
| MM157 | 0.344769 | 0.232538 | 0.076385 | 0.004077 | 0.045769 | 0.037 | 0.057 | 0.014769 | 0.002769 | 0.027 | 0.020077 | 0.014615 |
| MM3 | 0.446308 | 0.238077 | 0.046231 | 0.039231 | 0.021077 | 0.010923 | 0.000846 | 0.041077 | 7.69E-05 | 0.022077 | 0.041154 | 0.014538 |
| MM34 | 0.142769 | 0.066308 | 0.091692 | 0.366538 | 0.092385 | 0.038846 | 0.000231 | 0.024769 | 0.000154 | 0.008308 | 0.011385 | 0.015615 |
| MM67 | 0.239692 | 0.289923 | 0.062308 | 0.000615 | 0.036923 | 0.117769 | 0.046462 | 0.055231 | 7.69E-05 | 0.010846 | 0.008 | 0.009077 |
| MM84 | 0.273538 | 0.087846 | 0.153615 | 0.000231 | 0.055923 | 0.000154 | 0.047462 | 0.015462 | 0 | 0.046077 | 0.027462 | 0.040308 |
| MM122 | 0.315615 | 0.245462 | 0.078385 | 0.006308 | 0.019154 | 0.001769 | 0.050846 | 0.062462 | 7.69E-05 | 0.028077 | 0.047846 | 0.022538 |
| MM56 | 0.274308 | 0.171231 | 0.070923 | 0.001846 | 0.051462 | 0.187538 | 0.121231 | 0.001385 | 0.000462 | 0.013538 | 0.009154 | 0.013308 |
| MM19 | 0.256846 | 0.111077 | 0.071615 | 0.219615 | 0.060538 | 0.023154 | 7.69E-05 | 0.000308 | 0 | 0.027769 | 0.022308 | 0.033154 |
| MM132 | 0.274308 | 0.196846 | 0.095769 | 0.000308 | 0.032231 | 0.000769 | 0.045385 | 0.003769 | 0.000308 | 0.048308 | 0.054077 | 0.030615 |
| MM160 | 0.164692 | 0.167462 | 0.047385 | 0.282462 | 0.085462 | 0.02 | 0.009308 | 0.023615 | 0 | 0.025769 | 0.025462 | 0.014154 |
| MM59 | 0.253538 | 0.184538 | 0.120615 | 0.037615 | 0.054769 | 0.000923 | 0.096077 | 0.053154 | 0.000154 | 0.015 | 0.036 | 0.026769 |
| MM166 | 0.279846 | 0.134692 | 0.027692 | 0.359923 | 0.032462 | 0.029154 | 0.000154 | 0.005077 | 0.000231 | 0.017385 | 0.018846 | 0.017846 |
| MM21 | 0.242923 | 0.204923 | 0.054154 | 0.018692 | 0.061692 | 0.045923 | 0.115231 | 0.010538 | 0.080462 | 0.009692 | 0.007462 | 0.017154 |
| MM133 | 0.067077 | 0.375 | 0.070846 | 0.001615 | 0.042077 | 0.025 | 0.002923 | 0.000385 | 0.091846 | 0.030692 | 0.039231 | 0.015154 |
| MM31 | 0.163462 | 0.172385 | 0.215923 | 0.167923 | 0.067385 | 0.032538 | 0.000154 | 0.011154 | 0 | 0.019923 | 0.014 | 0.022923 |
| MM52 | 0.216923 | 0.077923 | 0.582 | 0.001923 | 0.015846 | 0.000308 | 0.027692 | 0.008615 | 0.000154 | 0.006231 | 0.010308 | 0.015923 |
| MM63 | 0.446692 | 0.107846 | 0.080923 | 0.121077 | 0.050308 | 0.034538 | 0.000308 | 0.036769 | 7.69E-05 | 0.008846 | 0.023077 | 0.011769 |
| MM81 | 0.422385 | 0.101769 | 0.064692 | 0.113615 | 0.024923 | 0.024769 | 7.69E-05 | 0.012154 | 0.000462 | 0.004615 | 0.021846 | 0.019154 |
| MM96 | 0.200769 | 0.168 | 0.026615 | 0.124538 | 0.078462 | 0.010308 | 0.003615 | 0.028 | 0 | 0.042923 | 0.021846 | 0.016 |
| MM106 | 0.247615 | 0.212154 | 0.078231 | 0.071615 | 0.037385 | 0.028846 | 0.010231 | 0.027692 | 0.003538 | 0.035846 | 0.068769 | 0.033923 |
| MM88 | 0.334385 | 0.205615 | 0.045385 | 0.016692 | 0.049 | 0.024154 | 0.023308 | 0.014231 | 0.104 | 0.019538 | 0.010385 | 0.016231 |
| MM147 | 0.309154 | 0.131769 | 0.048077 | 0.011385 | 0.047769 | 0.041923 | 0.101385 | 0.007308 | 0.017154 | 0.016308 | 0.020846 | 0.027231 |
| MM37 | 0.178231 | 0.063615 | 0.147538 | 0.255154 | 0.096231 | 0.002 | 0.001462 | 0.001538 | 0.002154 | 0.032231 | 0.006538 | 0.022692 |
| MM75 | 0.266538 | 0.255846 | 0.061308 | 0.005077 | 0.090615 | 0.002462 | 0.000692 | 0.070308 | 0.000308 | 0.027231 | 0.019538 | 0.020846 |
| MM91 | 0.526231 | 0.117538 | 0.065 | 0.029385 | 0.015154 | 0.006385 | 0.007 | 0.021846 | 0.000692 | 0.021385 | 0.037538 | 0.028231 |
| MM114 | 0.257615 | 0.041769 | 0.071308 | 0.001077 | 0.006231 | 0.000154 | 0.008308 | 0.014615 | 0 | 0.010615 | 0.012692 | 0.101615 |
| MM44 | 0.289231 | 0.194846 | 0.084308 | 0.000154 | 0.057308 | 0.100231 | 0.057231 | 0.073154 | 0 | 0.021154 | 0.010846 | 0.01 |
| MM87 | 0.243615 | 0.162154 | 0.027769 | 0.003846 | 0.164923 | 0.055692 | 0.117231 | 0.006462 | 0.075462 | 0.006 | 0.018692 | 0.006923 |
| MM130 | 0.464538 | 0.078154 | 0.062154 | 0.100154 | 0.052923 | 0.015077 | 0.032846 | 0.006769 | 0.050769 | 0.018385 | 0.008462 | 0.014846 |
| MM144 | 0.315615 | 0.133615 | 0.066 | 0.000385 | 0.032769 | 0.209846 | 0.003538 | 0.000231 | 0.021846 | 0.008077 | 0.003846 | 0.005846 |
| MM161 | 0.225692 | 0.230308 | 0.070692 | 0.055538 | 0.046231 | 0.067385 | 0.094538 | 0.034923 | 0.000154 | 0.024923 | 0.012308 | 0.012846 |
| MM58 | 0.261769 | 0.164308 | 0.227154 | 0.000154 | 0.048846 | 0.001077 | 0.063231 | 0.012615 | 0 | 0.027231 | 0.034077 | 0.023923 |
| MM110 | 0.357 | 0.170923 | 0.058 | 0.067308 | 0.044231 | 0.024462 | 0.012308 | 0.093846 | 0.004692 | 0.019692 | 0.035538 | 0.014385 |
| MM60 | 0.083538 | 0.061846 | 0.019462 | 0.449308 | 0.094154 | 0.039231 | 0.065154 | 0.005923 | 0.085462 | 0.016 | 0.006615 | 0.004769 |
| MM2 | 0.426462 | 0.143692 | 0.058154 | 0.125077 | 0.043077 | 0.018846 | 0.003077 | 0.020615 | 0.000385 | 0.021769 | 0.023077 | 0.019692 |
| MM146 | 0.282923 | 0.056615 | 0.085231 | 0.125154 | 0.015923 | 0.008308 | 0.040538 | 0.072231 | 0.081462 | 0.024077 | 0.014 | 0.026846 |
| MM112 | 0.261846 | 0.198154 | 0.068538 | 0.073692 | 0.090923 | 0.024846 | 7.69E-05 | 0.037692 | 0 | 0.028692 | 0.035769 | 0.026538 |
| MM125 | 0.346846 | 0.089308 | 0.082923 | 0.166385 | 0.051 | 0.028385 | 0 | 0.000538 | 7.69E-05 | 0.032231 | 0.020077 | 0.014846 |
| MM136 | 0.197615 | 0.145308 | 0.061154 | 0.146154 | 0.066077 | 0.017308 | 0.000154 | 0.079154 | 0.000154 | 0.031308 | 0.030077 | 0.016154 |
| MM142 | 0.389769 | 0.209769 | 0.072846 | 0.019 | 0.039385 | 0.003 | 0.007154 | 0.081154 | 0.008769 | 0.029769 | 0.020846 | 0.018 |
| MM38 | 0.159692 | 0.219231 | 0.090538 | 0.000538 | 0.077692 | 0.052385 | 0.015846 | 7.69E-05 | 0.154846 | 0.007923 | 0.015923 | 0.016615 |
| MM76 | 0.211769 | 0.132231 | 0.081769 | 0.270077 | 0.052308 | 0.028462 | 0.002846 | 0.020846 | 0.002077 | 0.011923 | 0.044231 | 0.028231 |
| MM113 | 0.403308 | 0.160846 | 0.052231 | 0.115385 | 0.020769 | 0.020308 | 0 | 0.046692 | 0 | 0.012538 | 0.039923 | 0.026538 |
| MM149 | 0.246231 | 0.176385 | 0.052769 | 0.109231 | 0.068462 | 0.024692 | 0.001385 | 0.009923 | 0 | 0.035385 | 0.026538 | 0.023462 |
| MM20 | 0.218615 | 0.087308 | 0.071231 | 0.001692 | 0.041385 | 0.048154 | 0.167077 | 0.096462 | 0.100615 | 0.007 | 0.009462 | 0.012923 |
| MM54 | 0.064538 | 0.206846 | 0.064462 | 0.000538 | 0.076308 | 0.121 | 0.024154 | 0.027538 | 0.135 | 0.015692 | 0.007769 | 0.010615 |
| MM57 | 0.242538 | 0.176 | 0.086846 | 0.000308 | 0.029769 | 0.185769 | 0.113692 | 0.004692 | 0.000385 | 0.010308 | 0.009615 | 0.014154 |
| MM111 | 0.311769 | 0.152692 | 0.060769 | 0.086769 | 0.060538 | 0.028308 | 0.011154 | 0.045 | 0.005231 | 0.020846 | 0.048692 | 0.027 |
| MM47 | 0.343692 | 0.166692 | 0.099308 | 0.000538 | 0.043 | 0.119077 | 0.063769 | 0.002538 | 0 | 0.011538 | 0.009077 | 0.015615 |
| MM89 | 0.277154 | 0.217231 | 0.062 | 0.001538 | 0.039462 | 0.054923 | 0.048231 | 0.001154 | 0.085308 | 0.023154 | 0.009769 | 0.017615 |
| MM158 | 0.260308 | 0.228462 | 0.078923 | 0.026846 | 0.056308 | 0.025923 | 0.045308 | 0.036154 | 0.073923 | 0.022462 | 0.017385 | 0.011692 |
| MM14 | 0.219846 | 0.177 | 0.189077 | 0.011077 | 0.029692 | 0 | 0.064 | 0.115385 | 7.69E-05 | 0.044308 | 0.026692 | 0.020231 |
| MM32 | 0.26 | 0.078769 | 0.038923 | 0.274385 | 0.098846 | 0.024 | 0.000154 | 0.000462 | 0.000231 | 0.013308 | 0.008615 | 0.015231 |
| MM40 | 0.154538 | 0.149231 | 0.079846 | 0.266846 | 0.112615 | 0.017846 | 0.001154 | 0.004615 | 0.002769 | 0.009923 | 0.012385 | 0.013769 |
| MM73 | 0.220231 | 0.163769 | 0.065077 | 0.001231 | 0.084538 | 0.050769 | 0.100462 | 0.040615 | 0.064154 | 0.016462 | 0.017308 | 0.018846 |
| MM100 | 0.217308 | 0.296538 | 0.062077 | 0.046615 | 0.071077 | 0.003462 | 0.002692 | 0.085231 | 0.006462 | 0.047154 | 0.007769 | 0.014308 |
| MM7 | 0.160846 | 0.325692 | 0.121769 | 0.000385 | 0.019231 | 0.023077 | 0.013308 | 0.000538 | 0.090615 | 0.032077 | 0.022 | 0.020308 |
| MM22 | 0.245077 | 0.047615 | 0.020846 | 7.69E-05 | 0.025 | 0.153846 | 0.255077 | 0.011462 | 0.201923 | 0.019846 | 0.000692 | 0 |
| MM62 | 0.169692 | 0.144846 | 0.302231 | 0.005462 | 0.036692 | 0.032154 | 0.041385 | 0.031769 | 0.002308 | 0.028692 | 0.024462 | 0.017692 |
| MM35 | 0.131769 | 0.095308 | 0.135846 | 0.289154 | 0.098692 | 0.019538 | 0.000385 | 0.050308 | 0.000615 | 0.017769 | 0.011692 | 0.015462 |
| MM53 | 0.225846 | 0.242231 | 0.137538 | 0.000462 | 0.052077 | 0.000615 | 0.051231 | 0.009615 | 0.000462 | 0.068462 | 0.048538 | 0.026 |
| MM82 | 0.272077 | 0.191 | 0.069385 | 0.096 | 0.029538 | 0.046923 | 0.012462 | 0.050077 | 0.000231 | 0.023769 | 0.036462 | 0.030538 |
| MM118 | 0.218077 | 0.274154 | 0.121692 | 0.002154 | 0.024615 | 0.001615 | 0.024769 | 0.006769 | 0.001462 | 0.073692 | 0.081308 | 0.028462 |
| MM154 | 0.279615 | 0.180923 | 0.085154 | 0.086385 | 0.049077 | 0.004231 | 0.000154 | 0.018077 | 0.000231 | 0.032692 | 0.03 | 0.042154 |

**Table S1C.** Meta-analysis of microbiomes of marmosets and other primates: information from the 1,418 fecal samples analyzed from 26 primate species.

| Group | Species | Family | Phylogeny | Sample size | Lifestyle | Diet Preference | |
| --- | --- | --- | --- | --- | --- | --- | --- |
| 1 | Infant USA ([1](#_ENREF_1)) | Hominidae | Hominid | 48 | Human, < 3 years | | Omnivore |
| 2 | Infant Malawian ([1](#_ENREF_1)) | Hominidae | Hominid | 62 | Human, < 3 years | | Omnivore |
| 3 | Infant Amazonas ([1](#_ENREF_1)) | Hominidae | Hominid | 29 | Human, < 3 years | | Omnivore |
| 4 | Human USA ([1](#_ENREF_1)) | Hominidae | Hominid | 266 | Human (Urban), adult (Most of them) | | Omnivore (western diet) |
| 5 | Human Malawian ([1](#_ENREF_1)) | Hominidae | Hominid | 52 | Human (Rural), adult (Most of them) | | Omnivore (traditional diet) |
| 6 | Human Amazonas ([1](#_ENREF_1)) | Hominidae | Hominid | 71 | Human (Preindustrial), adult (Most of them) | | Omnivore (traditional diet) |
| 7 | Chimpanzee ([2](#_ENREF_2)) | Hominidae | Hominid | 69 | Wild | | Herbivore |
| 8 | Orangutan ([3](#_ENREF_3)) | Hominidae | Hominid | 3 | Captive | | Herbivore |
| 9 | Western lowland gorilla ([3](#_ENREF_3)) | Hominidae | Hominid | 4 | Captive | | Herbivore |
| 10 | De Brazza’s monkey ([3](#_ENREF_3)) | Cercopithecidae | Old World monkey | 2 | Captive | | Omnivore |
| 11 | Hamadryas baboon([4](#_ENREF_4)) | Cercopithecidae | Old World monkey | 64 | Wild | | Omnivore |
| 12 | Red-shanked douc ([3](#_ENREF_3), [5](#_ENREF_5)) | Cercopithecidae | Old World monkey | 64 | Captive | | Herbivore |
| 12 | Red-shanked douc ([3](#_ENREF_3), [5](#_ENREF_5)) | Cercopithecidae | Old World monkey | 75 | Wild | | Herbivore |
| 13 | Francois langur ([6](#_ENREF_6)) | Cercopithecidae | Old World monkey | 3 | Wild | | Herbivore |
| 13 | Francois langur ([6](#_ENREF_6)) | Cercopithecidae | Old World monkey | 2 | Captive | | Herbivore |
| 14 | Javan langur ([5](#_ENREF_5)) | Cercopithecidae | Old World monkey | 22 | Captive | | Herbivore |
| 15 | Proboscis monkey ([5](#_ENREF_5)) | Cercopithecidae | Old World monkey | 32 | Captive | | Herbivore |
| 16 | Purple faced langur ([5](#_ENREF_5)) | Cercopithecidae | Old World monkey | 6 | Captive | | Herbivore |
| 17 | Black white colobus ([5](#_ENREF_5), [6](#_ENREF_6)) | Cercopithecidae | Old World monkey | 6 | Captive | | Herbivore |
| 18 | Guizhou snub nosed monkey ([5](#_ENREF_5), [6](#_ENREF_6)) | Cercopithecidae | Old World monkey | 12 | Wild | | Herbivore |
| 18 | Guizhou snub nosed monkey ([5](#_ENREF_5), [6](#_ENREF_6)) | Cercopithecidae | Old World monkey | 30 | Captive | | Herbivore |
| 19 | Sichuan snub nosed monkey ([5](#_ENREF_5), [6](#_ENREF_6)) | Cercopithecidae | Old World monkey | 15 | Captive | | Herbivore |
| 20 | Yunnan snub nosed monkey ([6](#_ENREF_6)) | Cercopithecidae | Old World monkey | 10 | Captive | | Herbivore |
| 21 | Tibetan macaque ([5](#_ENREF_5)) | Cercopithecidae | Old World monkey | 6 | Wild | | Omnivore |
| 22 | Rhesus macaque ([7](#_ENREF_7), [8](#_ENREF_8)) | Cercopithecidae | Old World monkey | 65 | Captive | | Omnivore |
| 23 | Common marmoset (this study) | Callitrichidae | New World monkey | 131 | Captive | | Omnivore |
| 24 | Geoffroy’s tamarin ([3](#_ENREF_3)) | Callitrichidae | New World monkey | 2 | Captive | | Omnivore |
| 25 | Emperor tamarin ([3](#_ENREF_3)) | Callitrichidae | New World monkey | 10 | Captive | | Omnivore |
| 26 | White-faced saki ([3](#_ENREF_3)) | Pitheciidae | New World monkey | 5 | Captive | | Omnivore |
| 27 | Spider monkey ([3](#_ENREF_3), [5](#_ENREF_5), [6](#_ENREF_6)) | Atelidae | New World monkey | 156 | Captive | | Omnivore |
| 28 | Mantled howling monkey ([3](#_ENREF_3)) | Atelidae | New World monkey | 51 | Wild | | Herbivore |
| 28 | Mantled howling monkey ([3](#_ENREF_3)) | Atelidae | New World monkey | 5 | Captive | | Herbivore |
| 29 | Blue-eyed black lemur ([3](#_ENREF_3)) | Lemuridae | Prosimian | 2 | Captive | | Omnivore |
| 30 | Ring-tailed lemur ([9](#_ENREF_9)) | Lemuridae | Prosimian | 18 | Wild | | Omnivore |
| 31 | Verreaux’s sifaka ([10](#_ENREF_10)) | Indriidae | Prosimian | 20 | Wild | | Herbivore |
