# Supplementary material for: Captive Common Marmosets (Callithrix jacchus) Are Colonized throughout Their Lives by a Community of Bifidobacterium Species with Species-Specific Genomic Content That Can Support Adaptation to Distinct Metabolic Niches
Source: mBio. 2021 Aug 3;12(4):e01153-21. doi: 10.1128/mBio.01153-21 (PMC8406136; doi:10.1128/mBio.01153-21)
Supplement: FIG S1 [file mbio.01153-21-sf001.pdf]

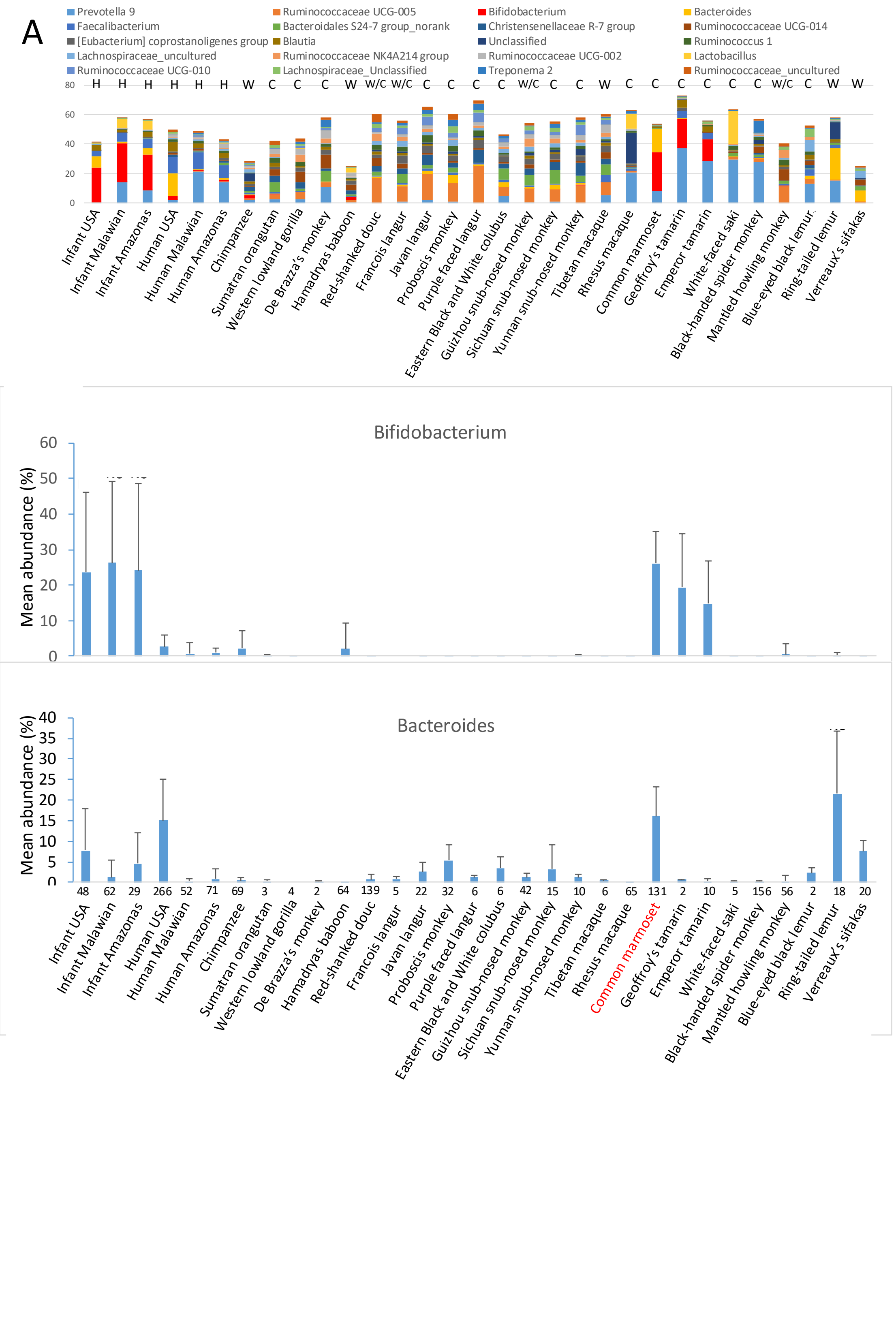
A
Prevotella 9
Ruminococcaceae UCG-005
Bifidobacterium
Bacteroides
Faecalibacterium
Bacteroidales S24-7 group_norank
Christensenellaceae R-7 group
Ruminococcaceae UCG-014
[Eubacterium] coprostanoligenes group
Blautia
Unclassified
Ruminococcus 1
Lachnospiraceae_uncultured
Ruminococcaceae NK4A214 group
Ruminococcaceae UCG-002
Lactobacillus
Ruminococcaceae UCG-010
Lachnospiraceae_Unclassified
Treponema 2
Ruminococcaceae_uncultured
H H H H H H W C C C W W/C W/C C C C C W/C C C W C C C C C C W/C C W W
Infant USA
Infant Malawian
Infant Amazonas
Human USA
Human Malawian
Human Amazonas
Chimpanzee
Sumatran orangutan
Western lowland gorilla
De Brazza's monkey
Hamadryas baboon
Red-shanked douc
Francois langur
Javan langur
Proboscis monkey
Purple faced langur
Eastern Black and White colubus
Guizhou snub-nosed monkey
Sichuan snub-nosed monkey
Yunnan snub-nosed monkey
Tibetan macaque
Rhesus macaque
Common marmoset
Geoffroy's tamarin
Emperor tamarin
White-faced saki
Black-handed spider monkey
Mantled howling monkey
Blue-eyed black lemur
Ring-tailed lemur
Verreaux's sifakas
Bifidobacterium
Mean abundance (%)
Bacteroides
Mean abundance (%)
48 62 29 266 52 71 69 3 4 2 64 139 5 22 32 6 6 42 15 10 6 65 131 2 10 5 156 56 2 18 20

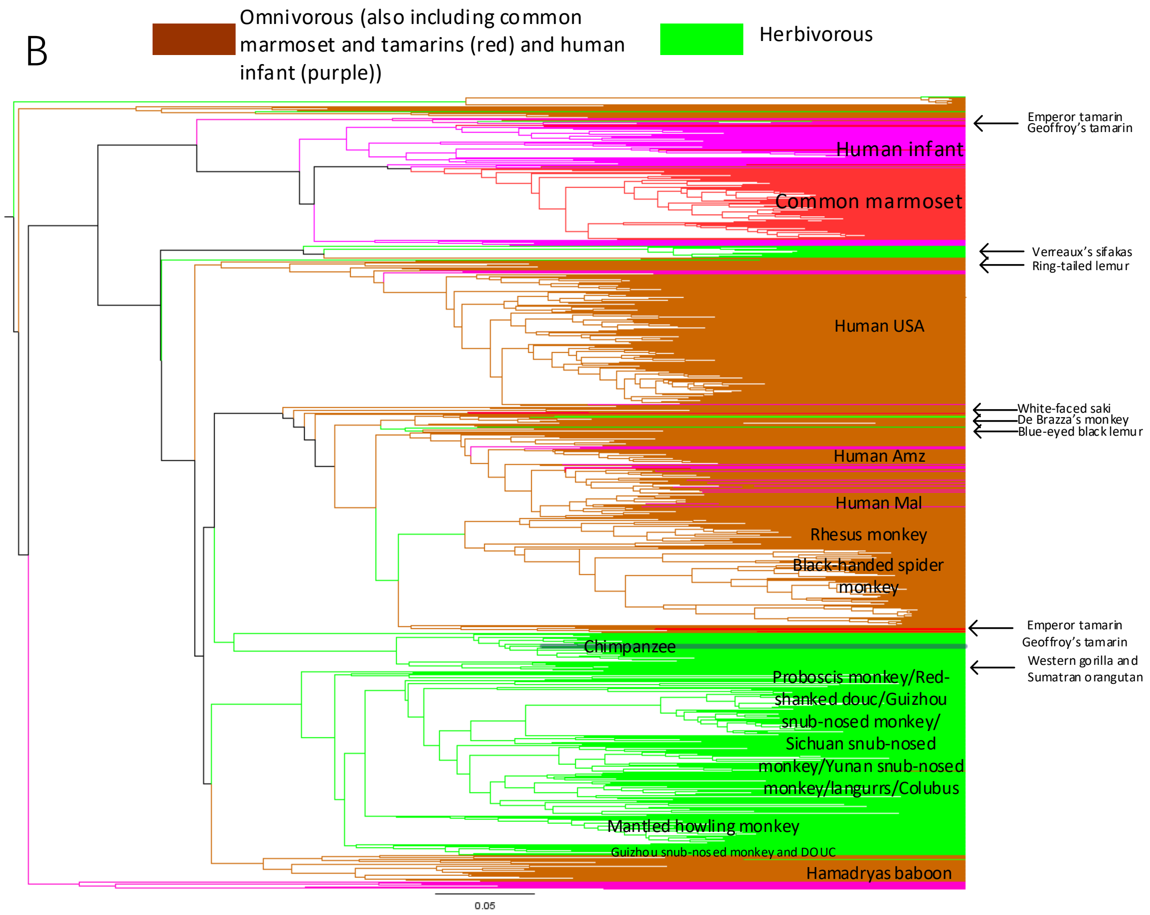
B
Omnivorous (also including common marmoset and tamarins (red) and human infant (purple))
Herbivorous
Emperor tamarin
Geoffroy's tamarin
Human infant
Common marmoset
Verreaux's sifakas
Ring-tailed lemur
Human USA
White-faced saki
De Brazza's monkey
Blue-eyed black lemur
Human Amz
Human Mal
Rhesus monkey
Black-handed spider monkey
Emperor tamarin
Geoffroy's tamarin
Chimpanzee
Western gorilla and Sumatran orangutan
Proboscis monkey/Red-shanked douc/Guizhou snub-nosed monkey/ Sichuan snub-nosed monkey/Yunan snub-nosed monkey/langurrs/Colubus
Mantled howling monkey
Guizhou snub-nosed monkey and DOUC
Hamadryas baboon
0.05

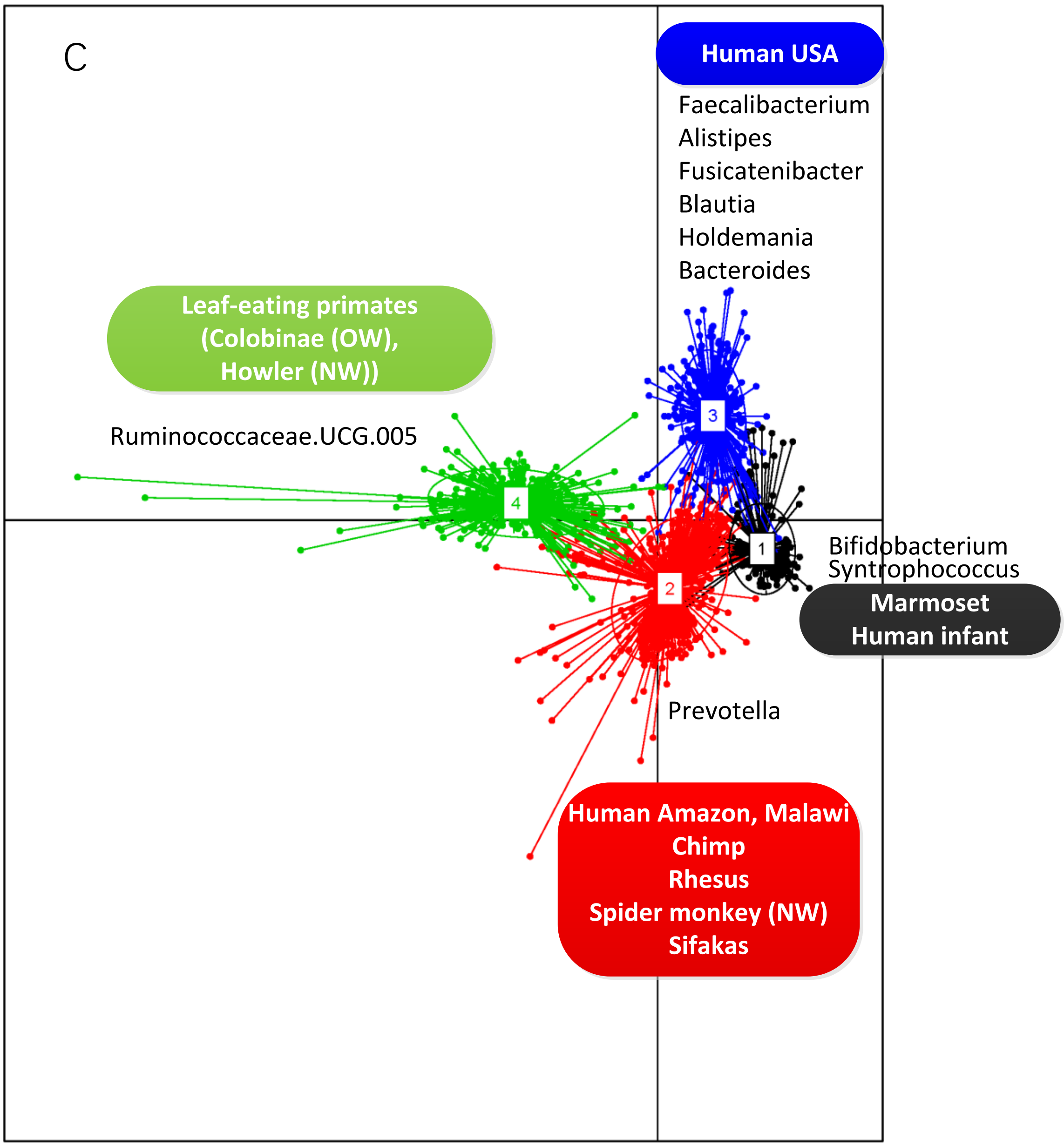
C
Human USA
Faecalibacterium
Alistipes
Fusicatenibacter
Blautia
Holdemania
Bacteroides
Leaf-eating primates
(Colobinae (OW),
Howler (NW))
Ruminococcaceae.UCG.005
3
4
1
2
Bifidobacterium
Syntrophococcus
Marmoset
Human infant
Prevotella
Human Amazon, Malawi
Chimp
Rhesus
Spider monkey (NW)
Sifakas

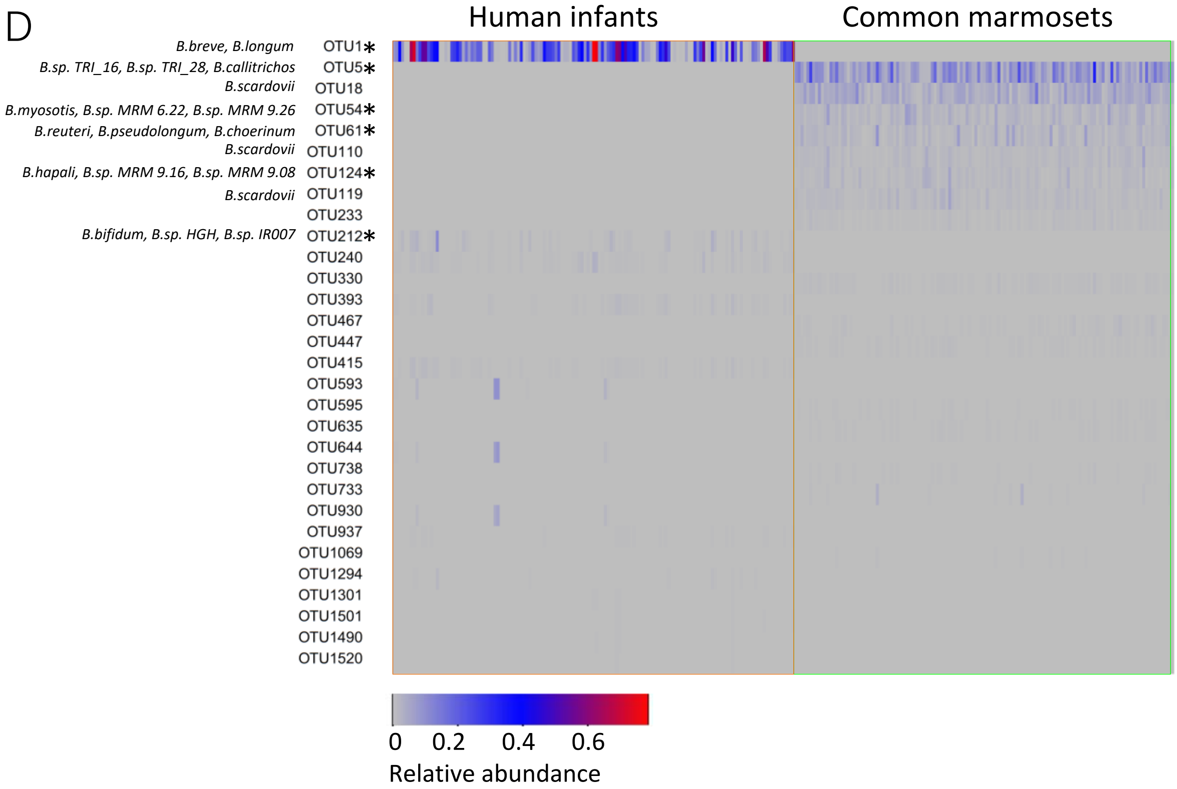

Figure S1 The gut microbiome analysis in the fecal samples of marmosets and other primates. (A) The gut microbiome composition in the fecal samples of marmosets and other primates. Dominant taxa present in the gut microbiome of 26 primate species are indicated with their mean relative abundances (bars) and standard deviation (whiskers). The top panel shows Taxa mean abundances in Captive (C), Wild (W), and Humans (H). In middle panels and bottom panel, mean abundances of *Bifidobacterium* (Panel B) and *Bacteroides* (Panel C) are shown for the same samples in the top panel. Numbers on the X-axis indicate the number of samples in the data set. (B) Hierarchical clustering of genus abundances representing 1,418 fecal microbiome samples from 26 primate species. Clustering was based on the Bray-Curtis distance (using genera relative abundances per sample) among 1,418 fecal microbiome. Amz, Amazonas. Mal, Malawian. (C) Partitioning around medoids (PAM) clustering algorithm of genus abundance from 1,418 fecal samples from 26 primate species. (D) Dominant *Bifidobacterium*

OTUs in adult common marmosets and human infants gut microbiomes. Representative reads of V4 16S rDNA amplicons from OTUs assigned to the genus Bifidobacterium by QiiME 2 were used in in BLAST searches to identify potential species. The top BLAST hits for species/subspecies of *Bifidobacterium* are indicated at the left for each OTU. No species are listed for OTUs lacking significant hit patterns to known *Bifidobacterium* species. The OTUs were heamapped for their relative abundances in human or the 131 adult marmoset samples from the CRC corresponding to the legend at bottom of Figure.
