# Supplementary material for: Captive Common Marmosets (Callithrix jacchus) Are Colonized throughout Their Lives by a Community of Bifidobacterium Species with Species-Specific Genomic Content That Can Support Adaptation to Distinct Metabolic Niches
Source: mBio. 2021 Aug 3;12(4):e01153-21. doi: 10.1128/mBio.01153-21 (PMC8406136; doi:10.1128/mBio.01153-21)
Supplement: FIG S2 [file mbio.01153-21-sf002.docx]

**Figure S2 Plate count enumeration of total *Bifidobacterium* from seven adult marmosets at three timepoints over a 13-day window**. Fecal samples were diluted and plated in triplicate onto *Bifidobacterium* selective media for each time point and enumerated. Averages of the triplicate counts from each time point are plotted on log10 scale (Y-axis) with the timepoints (dates) corresponding to the three different colors.
