# Supplementary material for: Captive Common Marmosets (Callithrix jacchus) Are Colonized throughout Their Lives by a Community of Bifidobacterium Species with Species-Specific Genomic Content That Can Support Adaptation to Distinct Metabolic Niches
Source: mBio. 2021 Aug 3;12(4):e01153-21. doi: 10.1128/mBio.01153-21 (PMC8406136; doi:10.1128/mBio.01153-21)
Supplement: TABLE S2 [file mbio.01153-21-st002.docx]

**Table S2 The Bifidobacterium analysis in this study.**

**Table S2A**  16S rRNA NCBI BLAST results from 116 isolates obtained from common marmoset fecal samples.

| SampleID | Media^a^ | | Age  (Days) | Individual | SequenceID | Whole Genome^b^ | NCBI BLAST best hit | BLAST accession | % Identity |
| --- | --- | --- | --- | --- | --- | --- | --- | --- | --- |
| MM203 | M5 | 1020 | | Tank | A05-047 | 8M5 | *Bifidobacterium aesculapii* | PRJDB4290 | 97% |
| MM68 | B4 | | 2388 | Athena | A04 |  | *Bifidobacterium aesculapii strain MRM* | NR_133981 | 98% |
| MM171 | B1 | | 2795 | Fabio | A11 |  | *Bifidobacterium aesculapii strain MRM 3/1* | NR_133981 | 98% |
| MM68 | B3 | | 2388 | Athena | F05 |  | *Bifidobacterium aesculapii strain MRM 3/1* | NR_133981 | 98% |
| MM45 | B4 | | 2700 | Bello | D11 |  | *Bifidobacterium aesculapii strain MRM 3/1* | NR_133981 | 98% |
| MM137 | B2 | | 2713 | Dexter | D05 |  | *Bifidobacterium aesculapii strain MRM 3/1* | NR_133981 | 99% |
| MM3 | B8 | | 2795 | Fabio | H02 |  | *Bifidobacterium aesculapii strain MRM 3/1* | NR_133981 | 98% |
| MM3 | M9 | | 2795 | Fabio | B03 | 10M9 | *Bifidobacterium aesculapii strain MRM 3/1* | NR_133981 | 98% |
| MM203 | B6 | | 1020 | Tank | D12 | 8B6 | *Bifidobacterium aesculapii strain MRM 4/2* | KC807990 | 98% |
| MM203 | M5 | | 1020 | Tank | G02 |  | *Bifidobacterium aesculapii strain MRM 4/2* | KC807990 | 99% |
| MM231 | B6 | | 2412 | Odie | E12 | 9B6 | *Bifidobacterium callitrichos strain AFB22-5* | NR_113172 | 99% |
| MM231 | B8 | | 2412 | Odie | G9 |  | *Bifidobacterium callitrichos strain AFB22-5* | NR_113172 | 99% |
| MM231 | B9 | | 2412 | Odie | G8 |  | *Bifidobacterium callitrichos strain AFB22-5* | NR_113172 | 99% |
| MM203 | M9 | | 1020 | Tank | C02 |  | *Bifidobacterium callitrichos strain AFB22-5* | NR_113172 | 98% |
| MM203 | B4 | | 1020 | Tank | B08 | 8B4 | *Bifidobacterium hapali strain MRM_8.14* | NR_147762 | 99% |
| MM45 | M3 | | 2700 | Bello | H11 | 4M3 | *Bifidobacterium kashiwanohense* | AP012327 | 96% |
| MM91 | M8 | | 2281 | Liilu | 3E3 |  | *Bifidobacterium lemurum* | NR_135862 | 97% |
| MM172 | B4 | | 2388 | Athena | 3C4 |  | *Bifidobacterium longum* | AP010890 | 91% |
| MM172 | B8 | | 2388 | Athena | 3C3 | 5B8 | *Bifidobacterium longum* | AP010890 | 91% |
| MM231 | B7 | | 2412 | Odie | 3G1 |  | *Bifidobacterium longum* | AP010890 | 91% |
| MM45 | B3 | | 2700 | Bello | H12 |  | *Bifidobacterium longum subsp. infantis 157F* | AP010890 | 98% |
| MM68 | B6 | | 2388 | Athena | E05 |  | *Bifidobacterium longum subsp. suis strain VB-5/9KY705021* | KY705021.1 | 99% |
| MM172 | B2 | | 2388 | Athena | D06 | 5B2 | *Bifidobacterium merycicum gene for 16S* | LC071847 | 97% |
| MM231 | B2 | | 2412 | Odie | 3A4 | 9B2 | *Bifidobacterium myosotis strain MRM_5.9* | NR_147760 | 99% |
| MM45 | B6 | | 2700 | Bello | E04 | 4B6 | *Bifidobacterium pseudocatenulatum strain* | JQ805694 | 97% |
| MM45 | M8 | | 2700 | Bello | E06 |  | *Bifidobacterium pseudocatenulatum strain* | JQ805694 | 98% |
| MM203 | B9 | | 1020 | Tank | H08 | 8B9 | *Bifidobacterium pullorum* | LC071802 | 97% |
| MM202 | M4 | | 2281 | Liilu | C02-012 |  | *Bifidobacterium reuteri strain* | NZ_JGZK00000000 | 99% |
| MM202 | M9 | | 2281 | Liilu | B02-014 |  | *Bifidobacterium reuteri strain* | NZ_JGZK00000000 | 99% |
| MM172 | B1 | | 2388 | Athena | D09 |  | *Bifidobacterium reuteri strain AFB22-1* | NR_113313 | 98% |
| MM68 | B1 | | 2388 | Athena | C07 |  | *Bifidobacterium reuteri strain AFB22-1* | NR_113313 | 98% |
| MM68 | B2 | | 2388 | Athena | E08 |  | *Bifidobacterium reuteri strain AFB22-1* | NR_113313 | 98% |
| MM172 | B5 | | 2388 | Athena | B01 |  | *Bifidobacterium reuteri strain AFB22-1* | NR_113313 | 99% |
| MM172 | B6 | | 2388 | Athena | B06 |  | *Bifidobacterium reuteri strain AFB22-1* | NR_113313 | 99% |
| MM172 | M2 | | 2388 | Athena | C10 |  | *Bifidobacterium reuteri strain AFB22-1* | NR_113313 | 99% |
| MM172 | M6 | | 2388 | Athena | D10 |  | *Bifidobacterium reuteri strain AFB22-1* | NR_113313 | 98% |
| MM68 | M6 | | 2388 | Athena | A06 | 3M6 | *Bifidobacterium reuteri strain AFB22-1* | NR_113313 | 97% |
| MM45 | B1 | | 2700 | Bello | D01 |  | *Bifidobacterium reuteri strain AFB22-1* | NR_113313 | 98% |
| MM45 | B7 | | 2700 | Bello | F04 | 4B7 | *Bifidobacterium reuteri strain AFB22-1* | NR_113313 | 98% |
| MM45 | B8 | | 2700 | Bello | H07 |  | *Bifidobacterium reuteri strain AFB22-1* | NR_113313 | 98% |
| MM45 | M2 | | 2700 | Bello | C09 |  | *Bifidobacterium reuteri strain AFB22-1* | NR_113313 | 99% |
| MM137 | B5 | | 2713 | Dexter | E02 |  | *Bifidobacterium reuteri strain AFB22-1* | NR_113313 | 99% |
| MM171 | B2 | | 2795 | Fabio | 3D3 |  | *Bifidobacterium reuteri strain AFB22-1* | NR_113313 | 99% |
| MM171 | B6 | | 2795 | Fabio | G5 |  | *Bifidobacterium reuteri strain AFB22-1* | NR_113313 | 98% |
| MM171 | B8 | | 2795 | Fabio | G12 |  | *Bifidobacterium reuteri strain AFB22-1* | NR_113313 | 98% |
| MM171 | M2 | | 2795 | Fabio | C08 | 6M2 | *Bifidobacterium reuteri strain AFB22-1* | NR_113313 | 98% |
| MM171 | M3 | | 2795 | Fabio | B10 |  | *Bifidobacterium reuteri strain AFB22-1* | NR_113313 | 99% |
| MM171 | M7 | | 2795 | Fabio | A08 |  | *Bifidobacterium reuteri strain AFB22-1* | NR_113313 | 99% |
| MM202 | B1 | | 2281 | Liilu | H06 |  | *Bifidobacterium reuteri strain AFB22-1* | NR_113313 | 94% |
| MM202 | B4 | | 2281 | Liilu | A12 |  | *Bifidobacterium reuteri strain AFB22-1* | NR_113313 | 99% |
| MM202 | B9 | | 2281 | Liilu | B05 |  | *Bifidobacterium reuteri strain AFB22-1* | NR_113313 | 99% |
| MM202 | M2 | | 2281 | Liilu | 3H3 |  | *Bifidobacterium reuteri strain AFB22-1* | NR_113313 | 99% |
| MM91 | M6 | | 2281 | Liilu | C03 |  | *Bifidobacterium reuteri strain AFB22-1* | NR_113313 | 99% |
| MM231 | B4 | | 2412 | Odie | C11 |  | *Bifidobacterium reuteri strain AFB22-1* | NR_113313 | 99% |
| MM203 | B2 | | 1020 | Tank | B07 |  | *Bifidobacterium reuteri strain AFB22-1* | NR_113313 | 99% |
| MM203 | B3 | | 1020 | Tank | 3C1 | 8B3 | *Bifidobacterium reuteri strain AFB22-1* | NR_113313 | 94% |
| MM203 | B3 | | 1020 | Tank | E07 |  | *Bifidobacterium reuteri strain AFB22-1* | NR_113313 | 89% |
| MM203 | M2 | | 1020 | Tank | G11 |  | *Bifidobacterium reuteri strain AFB22-1* | NR_113313 | 98% |
| MM203 | M7 | | 1020 | Tank | C05 |  | *Bifidobacterium reuteri strain AFB22-1* | NR_113313 | 99% |
| MM68 | B5 | | 2388 | Athena | F09 |  | *Bifidobacterium reuteri strain AFB22-1* | NR_113313 | 98% |
| MM172 | B9 | | 2388 | Athena | 3B3 | 5B9 | *Bifidobacterium saeculare* | LC071849 | 96% |
| MM68 | B9 | | 2388 | Athena | F06 | 3B9 | *Bifidobacterium saeculare / Bifidobacterium pullorum* | LC071849 | 96% |
| MM172 | B3 | | 2388 | Athena | B02 |  | *Bifidobacterium sp. MRM 6.22* | KP718950 | 99% |
| MM172 | M7 | | 2388 | Athena | F11 |  | *Bifidobacterium sp. MRM 6.22* | KP718950 | 99% |
| MM45 | B2 | | 2700 | Bello | D03 |  | *Bifidobacterium sp. MRM 6.22* | KP718950 | 98% |
| MM171 | M4 | | 2795 | Fabio | F10 |  | *Bifidobacterium sp. MRM 6.22* | KP718950 | 99% |
| MM91 | B9 | | 2281 | Liilu | F03 |  | *Bifidobacterium sp. MRM 8.12* | KP718947 | 99% |
| MM91 | B2 | | 2281 | Liilu | A10 |  | *Bifidobacterium sp. MRM 8.12* | KP718947 | 99% |
| MM231 | M4 | | 2412 | Odie | A05 |  | *Bifidobacterium sp. MRM 9.26* | KP718949 | 100% |
| MM231 | M9 | | 2412 | Odie | A01 |  | *Bifidobacterium sp. MRM 9.26* | KP718949 | 99% |
| MM45 | B9 | | 2700 | Bello | F02 |  | *Bifidobacterium sp. MRM 9.26/Bifidobacterium myosotis* | KP718949 | 99% |
| MM91 | B5 | | 2281 | Liilu | G01 | 1B5 | *Bifidobacterium sp. strain TRI_16* | KU298959 | 98% |

^a^Media used for isolation and plate number: M=MRS, B= BSIM

^b^Isolates used for whole genome sequencing

**Table S2B** Whole genome sequencing information for 18 *Bifidobacterium* strains isolated from 7 adult common marmosets.

| **Sample** | **Reads** | **Bases** | **Average Length** | **Depth** |
| --- | --- | --- | --- | --- |
| 1B5_S15 | 1,866,176 | 445,743,459 | 238.85 | 169.7 |
| 3B9_S14 | 1,470,524 | 351,578,059 | 239.08 | 135.4 |
| 3M6_S8 | 1,631,830 | 370,049,569 | 226.77 | 138.7 |
| 4B6_S16 | 3,808,160 | 776,169,165 | 203.82 | 295.1 |
| 4B7_S7 | 2,847,822 | 568,231,289 | 199.53 | 211.6 |
| 4M3_S21 | 2,235,084 | 516,647,352 | 231.15 | 214.3 |
| 5B2_S5 | 2,256,800 | 519,706,767 | 230.28 | 194.1 |
| 5B8_S4 | 2,041,352 | 473,085,830 | 231.75 | 174.3 |
| 5B9_S13 | 1,447,978 | 346,859,576 | 239.55 | 133.8 |
| 6M2_S9 | 2,415,132 | 506,993,658 | 209.92 | 178.2 |
| 8B3_S10 | 1,692,152 | 391,246,564 | 231.21 | 144.7 |
| 8B4_S2 | 1,571,778 | 356,431,040 | 226.77 | 121.3 |
| 8B6_S20 | 1,104,592 | 241,617,816 | 218.74 | 95.3 |
| 8B9_S17 | 1,806,496 | 401,681,600 | 222.35 | 158.2 |
| 8M5_S18 | 2,241,518 | 496,918,111 | 221.69 | 176.7 |
| 9B2_S6 | 1,314,988 | 302,248,589 | 229.85 | 92.8 |
| 9B6_S1 | 1,962,386 | 433,693,211 | 221 | 144.3 |
| 10M9_S19 | 2,200,598 | 513,483,481 | 233.34 | 204.8 |

**Table S2C** The assembly and annotation information of 18 marmoset Adult-origin *Bifidobacterium* strains

| **Strains** | **N50** | **Sequence Length** | **Number of CDSs** | **Average Protein Length** |
| --- | --- | --- | --- | --- |
| Marmoset_10M9_S19 | 110200 | 2506229 | 1942 | 366.2 |
| Marmoset_1B5_S15 | 130592 | 2622313 | 1994 | 377.8 |
| Marmoset_3B9_S14 | 157134 | 2595630 | 1988 | 378.8 |
| Marmoset_3M6_S8 | 153414 | 2667307 | 2069 | 363.4 |
| Marmoset_4B6_S16 | 73645 | 2630244 | 2075 | 362.6 |
| Marmoset_4B7_S7 | 115733 | 2685236 | 2122 | 351.7 |
| Marmoset_4M3_S21 | 147113 | 2410338 | 1879 | 370.5 |
| Marmoset_5B2_S5 | 291455 | 2677759 | 2082 | 363.2 |
| Marmoset_5B8_S4 | 331455 | 2714621 | 2113 | 362.2 |
| Marmoset_5B9_S13 | 130132 | 2591544 | 1985 | 377.4 |
| Marmoset_6M2_S9 | 113436 | 2845272 | 2220 | 359.5 |
| Marmoset_8B3_S10 | 90654 | 2703233 | 2085 | 364.4 |
| Marmoset_8B4_S2 | 332217 | 2937471 | 2320 | 356.8 |
| Marmoset_8B6_S20 | 83151 | 2535846 | 1952 | 366 |
| Marmoset_8B9_S17 | 90860 | 2538285 | 1977 | 362.8 |
| Marmoset_8M5_S18 | 148014 | 2811779 | 2190 | 364.2 |
| Marmoset_9B2_S6 | 76472 | 3255843 | 2520 | 350.7 |
| Marmoset_9B6_S1 | 93247 | 3005601 | 2239 | 369.5 |

CDSs, coding regions.

**Table S2D** qPCR primers for marmoset Bifidobacterium species

**Table S2E** Published *Bifidobacterium* genomes used in this study

| ***Bifdidobacterium* (B.) genomes** | **NCBI Aceesion number** |
| --- | --- |
| *B.actinocoloniiforme_DSM_22766* | CP011786.1 |
| *B.adolescentis_ATCC_15703* | AP009256.1 |
| *B.adolescentis_DSM_20087* | GCF_000702865.1 |
| *B.aesculapii_strain_DSM_26737* | NZ_BCFK01000003.1 |
| *B.angulatum_DSM_20098* | AP012322.1 |
| *B.animalis_subsp._lactis_AD011* | NC_011835.1 |
| *B.animalis_subsp._lactis_B1-4* | NC_012814.1 |
| *B.animalis_subsp._lactis_DSM_10140* | CP001606.1 |
| *B.animalis_subsp._LMG_10508* | GCF_000741485.1 |
| *B.aquikefiri_strain_LMG_28769* | NZ_MWXA01000005.1 |
| *B.asteroides_PRL2011* | CP003325.1 |
| *B.biavatii_DSM_23969* | GCF_000741165.1 |
| *B.bifidum_DSM_20456* | NZ_AP012323.1 |
| *B.bohemicum_DSM_22767* | NZ_JGYP01000002.1 |
| *B.bombi_DSM_19703* | NZ_ATLK01000001.1 |
| *B.boum_DSM_20432* | GCF_000771385.1 |
| *B.breve_DSM_20213* | AP012324.1 |
| *B.breve_NCFB_2258* | NZ_CP006714.1 |
| *B.breve_UCC2003* | NC_020517.1 |
| *B.callitrichos_DSM_23973* | NZ_JGYS01000001.1 |
| *B.catenulatum_DSM_16992* | AP012325.1 |
| *B.choerinum_DSM_20434* | GCF_000484675.1 |
| *B.commune_strain_R-52791* | NZ_FMBL01000001.1 |
| *B.coryneforme_DSM_20216* | GCF_000771345.1 |
| *B.crudilactis_Strain_LMG_10738* | GCF_000738005. |
| *B.cuniculi_strain_LMG_10738* | NZ_JGYV01000001.1 |
| *B.dentium_JCM_1195* | AP012326.1 |
| *B.eulemuris_strain_DSM_100216* | NZ_MWWZ01000004.1 |
| *B.gallicum_DSM_20093* | NZ_JGYW01000005.1 |
| *B.gallinarum_strain_LMG_11586* | NZ_JGYX01000001.1 |
| *B.hapali_strain_DSM_100202* | NZ_MWWY01000005.1 |
| *B.indicum_LMG_11587* | CP006018.1 |
| *B.kashiwanohense_DSM_21854* | NZ_AP012327.1 |
| *B.lemurum_strain_DSM_28807* | NZ_BDIS01000011.1 |
| *B.longum_DJO10A* | NC_010816.1 |
| *B.longum_JCM_1217* | AP010888.1 |
| *B.longum_JDM_301* | NC_014169.1 |
| *B.longum_NCC2705* | AE014295.3 |
| *B.longum_infantis_DSM_20088* | NC_011593.1 |
| *B.magnum_strain_LMG_11591* | NZ_JGZB01000004.1 |
| *B.merycicum_DSM_6492* | GCA_900129045.1 |
| *B.minimum_DSM_20102* | GCA_000421685.1 |
| *B.mongoliense_DSM_21395* | NZ_JGZE01000001.1 |
| *B.moukalabense_DSM_27321* | NZ_AZMV01000007.1 |
| *B.myosotis_strain_DSM_100196* | NZ_MWWW01000004.1 |
| *B.psedudolongum_DSM_20092* | GCF_000687595.1 |
| *B.pseudolongum_PV8-2* | CP007457.1 |
| *B.pseudocatenulatum_DSM_20438* | AP012330.1 |
| *B.pseudolongum_AGR2145* | GCF_000421365.1 |
| *B.psychraerophilum_DSM_22366* | GCF_000771565.1 |
| *B.pullorum_DSM_20433* | NZ_JDUI01000001.1 |
| *B.reuteri_DSM_23975* | NZ_JGZK01000003.1 |
| *B.ruminantium_DSM_6489* | NZ_JHWQ01000001.1 |
| *B.saeculare_DSM_6531 = LMG_14934* | GCF_000741375.1 |
| *B.saguini_DSM_23967* | NZ_JGZN01000003.1 |
| *B.scardovii_JCM_12489 (DSM_13734)* | AP012331.1 |
| *B.stellenboschense_DSM_23968* | GCF_000741785.1 |
| *B.stercoris_JCM_15918* | JGZQ01000008.1 |
| *B.subtile_DSM_20096* | GCF_000426405.1 |
| *B.thermacidophilum_DSM_17755* | GCF_000771045.1 |
| *B.thermophilum_DSM_20210* | GCF_000771265.1 |
| *B.thermophilum_DSM_20212* | GCF_000687575.1 |
| *B.thermophilum_RBL67* | CP004346.1 |
| *B.tissieri_strain_DSM_100201* | NZ_MWWV01000004.1 |
| *B.tsurumiense_JCM_13495* | GCF_000741765.1 |
