# Supplementary material for: Captive Common Marmosets (Callithrix jacchus) Are Colonized throughout Their Lives by a Community of Bifidobacterium Species with Species-Specific Genomic Content That Can Support Adaptation to Distinct Metabolic Niches
Source: mBio. 2021 Aug 3;12(4):e01153-21. doi: 10.1128/mBio.01153-21 (PMC8406136; doi:10.1128/mBio.01153-21)
Supplement: FIG S3 [file mbio.01153-21-sf003.docx]

**Figure S3 Xyloglucan islands present in the genomes of MARM_A1, *B. myosotis*, and *B. hapali***. Genomic islands that encode putative enzymes for degradation of xyloglucans are aligned from the genomes of MARM_A1, *B. myosotis*, and *B. hapali*. Genes encoding a putative ABC transport system for galacturonooligosaccharides are shaded in orange, including the *yteP*-like gene at the 5’ end. Putative esterase genes are colored light blue, the *afuC*-like α-fucosidases colored red, GH3 *bglX*-like β-glucosidases dark blue, GH5 *bglC*-like β-endoglucanases in blue, GH31 α-xylosidase light green, GH43 β-xylosidase dark green and GH42 β-galactanase in yellow.
