# Supplementary material for: Captive Common Marmosets (Callithrix jacchus) Are Colonized throughout Their Lives by a Community of Bifidobacterium Species with Species-Specific Genomic Content That Can Support Adaptation to Distinct Metabolic Niches
Source: mBio. 2021 Aug 3;12(4):e01153-21. doi: 10.1128/mBio.01153-21 (PMC8406136; doi:10.1128/mBio.01153-21)
Supplement: FIG S4 [file mbio.01153-21-sf004.docx]

**Figure S4 The XL-1 Xylan island unique to the genome of *B. reuteri* phylotype 1 strains**. The XL-1 xylan island from *B. reuteri* phylotype 1 strains is shown at top. The island is inserted adjacent to the xylose isomerase gene and the corresponding regions adjacent to the xylose isomerase gene are indicated below from the genomes of *B. myosotis*, *B. reuteri* phylotype 2, *B. hapali*, *B. aesculapii*, MARM_A2, MARM_A1, and MARM_A3. Genes in the *B. reuteri* phylotype 1 XL-1 island include *ugpB*, *ugpA*, and *ugpE*-like genes encoding components of a putative ABC transport system (orange), genes with GH domains characteristic of β-xylanases, β-xylosidases, and α-arabinases (shades of green), and the GH2-containing xylan esterase like gene (gray). Xylan-associated genes at this same position in the *B. myosotis* genome encode two GH43-domain XynB-like xylosidases/arabinases (shades of green) an AES-type esterase (light blue) and a putative acetyltransferase (blue).
