# Supplementary material for: Captive Common Marmosets (Callithrix jacchus) Are Colonized throughout Their Lives by a Community of Bifidobacterium Species with Species-Specific Genomic Content That Can Support Adaptation to Distinct Metabolic Niches
Source: mBio. 2021 Aug 3;12(4):e01153-21. doi: 10.1128/mBio.01153-21 (PMC8406136; doi:10.1128/mBio.01153-21)
Supplement: FIG S5 [file mbio.01153-21-sf005.docx]

**Figure S5 Arabinose operons from *Bifidobacterium* species isolated from callitrichids**. Genomic segments of the arabinose utilization operon and *hypBA2*-like genes are aligned from genome sequences of *B. aesculapii*, *B. parmae*, *B. primatium*, and *B. catulorum*. Gene IDs are indicated for the *B. aesculapii* 8B9 genomic sequence. The *araQ* regulatory gene is colored in red while orthologues of the *araDAB* catalytic genes are colored dark blue. The conserved *hypBA2*-like gene is colored light blue. The genomes of *B. parmae* and *B. primatium* contain genes encoding a putative Von Wildebrand factor domain (vWF gene, peach color) and an exonuclease (yellow) upstream of the *hypBA2*-like orthologues while the *B. catulorum* genome carries only the exonuclease like gene at that position.
